# Implications of size dispersion on X-ray scattering of crystalline nanoparticles: CeO_2_ as a case study

**DOI:** 10.1107/S1600576724003108

**Published:** 2024-05-31

**Authors:** Adriana Valério, Fabiane J. Trindade, Rafaela F. S. Penacchio, Bria Cisi, Sérgio Damasceno, Maurício B. Estradiote, Cristiane B. Rodella, Andre S. Ferlauto, Stefan W. Kycia, Sérgio L. Morelhão

**Affiliations:** a Institute of Physics, University of São Paulo, São Paulo, Brazil; b Laboratory of Materials for Energy, Engineering, Modelling and Applied Social Sciences Center, Federal University of ABC, Santo André, São Paulo, Brazil; c Brazilian Synchrotron Light Laboratory – SIRIUS/CNPEM, Campinas, São Paulo, Brazil; d Department of Physics, University of Guelph, Guelph, Ontario, Canada; HPSTAR and Harbin Institute of Technology, People’s Republic of China

**Keywords:** catalysts, ceria, nanocrystals, size dispersion, X-ray scattering

## Abstract

Controlling the shape and size dispersivity and crystallinity of nanoparticles (NPs) has been a challenge in identifying these parameters’ role in the physical and chemical properties of NPs. The need for reliable quantitative tools for analyzing the dispersivity and crystallinity of NPs is a considerable problem in optimizing scalable synthesis routes capable of controlling NP properties. The most common tools are electron microscopy (EM) and X-ray scattering techniques. However, each technique has different susceptibility to these parameters, implying that more than one technique is necessary to characterize NP systems with maximum reliability. Wide-angle X-ray scattering (WAXS) is mandatory to access information on crystallinity. In contrast, EM or small-angle X-ray scattering (SAXS) is required to access information on whole NP sizes. EM provides average values on relatively small ensembles in contrast to the bulk values accessed by X-ray techniques. Besides the fact that the SAXS and WAXS techniques have different susceptibilities to size distributions, SAXS is easily affected by NP–NP interaction distances. Because of all the variables involved, there have yet to be proposed methodologies for cross-analyzing data from two techniques that can provide reliable quantitative results of dispersivity and crystallinity. In this work, a SAXS/WAXS-based methodology is proposed for simultaneously quantifying size distribution and degree of crystallinity of NPs. The most reliable easy-to-access size result for each technique is demonstrated by computer simulation. Strategies on how to compare these results and how to identify NP–NP interaction effects underneath the SAXS intensity curve are presented. Experimental results are shown for cubic-like CeO_2_ NPs. WAXS size results from two analytical procedures are compared, line-profile fitting of individual diffraction peaks in opposition to whole pattern fitting. The impact of shape dispersivity is also evaluated. Extension of the proposed methodology for cross-analyzing EM and WAXS data is possible.

## Abstract 2

A methodology based on small- and wide-angle X-ray scattering is proposed for simultaneously quantifying size distribution and degree of crystallinity of nanoparticles. Experimental results are shown for cubic-like CeO_2_ nanoparticles.

## 1. Introduction

Metal and metal-oxide crystalline nanoparticles (NPs) have potential applications in many research areas due to unique physical and chemical properties related to their high surface-to-volume ratio (Grassian, 2008 ▸; Ramos-Guivar *et al.*, 2021 ▸; Canchanya-Huaman *et al.*, 2021 ▸). Accessing size distribution in NP systems is fundamental for understanding structure-dependent functionalities, morphology and other structural variables (Zhang *et al.*, 2004 ▸; Borchert *et al.*, 2005 ▸; Frenkel *et al.*, 2005 ▸; Keshari & Pandey, 2008 ▸; Schwung *et al.*, 2014 ▸; Schmidt *et al.*, 2016 ▸; Clarke *et al.*, 2018 ▸; Freitas Cabral *et al.*, 2020 ▸; Kabir *et al.*, 2022 ▸). In the case of ceria (CeO_2_) NPs, the reversible Ce^4+^/Ce^3+^ redox cycle is associated with the formation of oxygen vacancies in the crystalline structure, having a high capacity to store and release oxygen with excellent stability (Yang *et al.*, 2021 ▸; Xu *et al.*, 2021 ▸; Schilling *et al.*, 2018 ▸; Ziemba *et al.*, 2021 ▸). These properties make CeO_2_ one of the most promising catalysts in many reactions (Smith *et al.*, 2021*b* ▸; Sun *et al.*, 2016 ▸; Trovarelli & Llorca, 2017 ▸). By adjusting NP size and morphology, the performance of catalysts can improve significantly. The CeO_2_ NP morphology is tunable by controlling critical parameters such as pH, pressure and temperature in hydrothermal synthesis techniques (Smith *et al.*, 2021*b* ▸; Sun *et al.*, 2016 ▸; Mai *et al.*, 2005 ▸; Dong *et al.*, 2020 ▸; Chavhan *et al.*, 2020 ▸; Sreekanth *et al.*, 2019 ▸; Trindade *et al.*, 2022 ▸). Despite previous studies, the role of size dispersivity in catalytic performance requires further analysis, as the tool to quantify NP size dispersivity still needs to be improved. It is a general issue when analyzing nanopowders as the lack of reliable characterization techniques for determination of size distributions makes the stated NP properties questionable (Jensen *et al.*, 2006 ▸).

Small-angle X-ray scattering (SAXS) and X-ray diffraction (XRD) are tools widely used to access NP systems’ size information (Guinier, 1994 ▸; Zemb & Lindner, 2002 ▸; Sivia, 2011 ▸; Leoni, 2019 ▸). These are bulk techniques because X-rays interact with the illuminated sample volume, providing average values over large ensembles of NPs. Both techniques measure the angle-dependent distribution of the radiation scattered by atomic electrons in the sample. The difference is that SAXS techniques examine scattering angles below a few degrees, probing the average size and shape of NPs regardless of crystalline perfection, whereas XRD techniques examine wide angles where scattering is measurable at Bragg angles due to the periodicity of crystalline materials. Consequently, SAXS is susceptible to the entire volume of an NP, whereas XRD accesses only the crystallized portion of the NP’s volume. Using both SAXS and XRD techniques, or in more general terms, evaluating the X-ray scattering from small to wide angles, allows, in principle, the optimization of synthesizing processes aimed at controlling the size, size dispersion and crystallinity of the NPs.

Methodologies are available to access size-distribution information, whether for SAXS or XRD techniques [see, for instance, Bergeret & Gallezot (2008 ▸)]. Fitting the intensity curve by modeling particle shape, size distribution and interparticle interactions is currently the most common approach in analyzing SAXS data from polydisperse NPs (Glatter, 1980 ▸; Beaucage *et al.*, 2004 ▸; Jensen *et al.*, 2006 ▸; Borchert *et al.*, 2005 ▸; Garcia *et al.*, 2019 ▸). At wide angles, line-broadening analysis of diffraction peaks stands as the general approach to address size distribution. There is a very long discussion that can be traced back to the early decades of the 20th century about the actual role of size distribution in the line profile of the diffraction peaks (Jones, 1938 ▸; Bertaut, 1950 ▸; Le Bail & Louër, 1978 ▸; Langford & Louër, 1982 ▸; Langford *et al.*, 2000 ▸; Leonardi *et al.*, 2022 ▸). As particle dispersivity in powder samples has been seen more as a consequence of their preparation history than a physical property, most traditional methods of line-profile analysis are primarily concerned with effects arising from crystalline imperfections and instrumentation (Cheary & Coelho, 1992 ▸; Kril & Birringer, 1998 ▸; Snyder *et al.*, 1999 ▸; Coelho, 2000 ▸). Available methodologies for accessing size-distribution information consist of a secondary analysis of the peak profile (Warren & Averbach, 1950 ▸; Leoni, 2019 ▸) or the use of a more elaborate approach to account for size-distribution parameters when modeling diffraction patterns (Scardi & Leoni, 2001 ▸, 2002 ▸; Cervellino *et al.*, 2005 ▸; Scardi, 2020 ▸). Despite many developments in X-ray techniques to resolve size distribution, methodologies for combining results from SAXS and XRD techniques into systematic procedures, along with reliability criteria, to quantify the size dispersivity and crystallinity of NPs are still lacking.

In practice, attesting the accuracy and reliability of such meth­o­dologies is complicated because retrieving size-distribution parameters from X-ray patterns is ambiguous due to the countless size distributions capable of providing similar scattering intensity curves. Validation of methodologies by comparing size-distribution results from different techniques, such as XRD and electron microscopy (EM), is also tricky (Leoni & Scardi, 2004 ▸). XRD is a bulk technique that examines the coherent length of periodic structures (Morelhão *et al.*, 2019 ▸), *i.e.* the crystalline domain size, over a large ensemble of NPs. EM examines shape and size distributions over a much smaller ensemble (Williams & Carter, 2009 ▸). Both size distributions are compatible in systems of fully crystalline NPs with narrow size dispersion where a small ensemble is representative of the volume sampled by XRD (Scardi & Gelisio, 2016 ▸). Discrepancies with the size results obtained with SAXS, another bulk technique, can arise due to NP crystalline domains being smaller than their sizes, such as in typical core/shell NPs with a crystalline core and an amorphous shell (Ichikawa *et al.*, 2018 ▸). On top of this, the two techniques weight the size distribution differently, and this difference in weighting brings extra information capable of resolving the size dispersivity and crystallinity of the NPs (Morelhão & Kycia, 2022 ▸).

This work investigates the direct implications of size distribution in SAXS and XRD techniques through virtual systems of polydisperse NPs. In such well controlled systems, free of instrumental and crystalline imperfection effects, SAXS and XRD measurements demonstrate the most accessible and reliable quantitative pieces of information available about the size distribution. Initially, computed pair distance distribution functions (PDDFs) (Glatter, 1977 ▸; Liu & Zwart, 2012 ▸) for virtual NPs with sizes ranging from 1 to 90 nm lead to X-ray scattering patterns encompassing the entire angular range accessed by the SAXS and XRD techniques. After verifying that size information is available in the simulated patterns, we compute size-distribution effects by weighting the simulated patterns for monodisperse sizes with a lognormal size-distribution probability function. This provides a com­puta­tional demonstration that the scattering curve width around the direct beam and the Bragg peak widths are dictated by the medians of the size distribution’s sixth- and fourth-momentum integrals, respectively. On the basis of these quantities, we propose a SAXS/wide-angle X-ray scattering (WAXS) methodology for simultaneously quantifying size distribution and degree of crystallinity of NPs. Actual SAXS and XRD measurements analyze real samples: a series of powder samples of ceria NPs. The size-discrepant results from these measurements are used as a test of the proposed methodology, revealing additional sources of size discrepancies related to NP–NP interactions and possible strategies for comparing SAXS and XRD results. EM results are used to confirm the cubic-like morphology of the NPs and the existence of size dispersion in all samples. Detected changes in the size and size dispersivity related to synthesis parameters suggest analytical procedures to guide further optimization of chemical routes to control these properties in crystalline ceria NPs.

## 2. Theoretical basis

NP size and shape roles in SAXS and XRD techniques are accurately computed by the well known Debye scattering equation (Scardi & Gelisio, 2016 ▸), *e.g.* equation (1). This is valid in the entire angular region, from ultra-small to wide angles. The computational time cost is high for NPs with sizes above a few tens of nanometres. However, as accurate quantification is needed to illustrate the impact of size dispersivity properly, the Debye scattering equation is used here as follows.

### 2.1. Monodisperse systems

A system of *N* identical non-interacting NPs randomly oriented in space scatters X-rays according to *I*(*Q*) = *NI*_Th_ *P*(*Q*), where *I*_Th_ is the Thomson intensity scattered by a single electron accounting for polarization effects and 

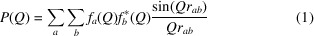

is the NP scattering power (Debye, 1915 ▸; Guinier, 1994 ▸; Gelisio *et al.*, 2010 ▸; Morelhão, 2016 ▸; Scardi & Gelisio, 2016 ▸). The term 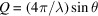 is the modulus of the scattering vector for a 2θ angle of scattering, *f*_*a*,*b*_(*Q*) is the atomic scattering factor of atom *a* or *b* with resonant amplitudes for X-rays of wavelength λ, and *r*_*ab*_ = |**r**_*a*_ − **r**_*b*_| are the distances between any pair of atoms at the **r**_*a*_ and **r**_*b*_ frozen-in-time positions; the indices *a* and *b* run over all the atoms in the NP.

The temporal coherence of X-ray beams is of the order of 1 fs. At this timescale, atomic thermal vibrations are relatively slow. Actual scattering patterns acquired over long periods correspond to the sum of the instantaneous intensities of statistically equivalent structures with displaced atoms within their vibration amplitudes. Powder samples of identical NPs represent ensembles of such statistically equivalent structures. Random uncorrelated displacements are commonly accounted for in the Debye–Waller factors (Scardi & Gelisio, 2016 ▸), while collective vibrations – phonons – are responsible for thermal diffuse scattering (Als-Nielsen & McMorrow, 2011 ▸). For this work, random displacements d**r** around the average position 〈**r**_*a*_〉 were considered for each atom, as in **r**_*a*_ = 〈**r**_*a*_〉 + d**r**. The scattering power of a frozen NP with random displacements is practically the same as the average scattering power computed over the ensemble of statistically equivalent structures; see a demonstration in Section S1 of the supporting information.

At small scattering angles where the resolution is insufficient to resolve the shortest atomic distances inside the NP, the scattering power 

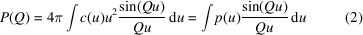

involves the electron–electron pair correlation function *c*(*u*) = ρ_s_(*r*) 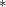 ρ_s_(−*r*) (Guinier, 1994 ▸), equivalent to the Patterson function for crystals (Patterson, 1935 ▸). ρ_s_(*r*) is a spherically symmetric electron-density function representing the NP in a system of identical NPs randomly oriented in space (Morelhão, 2016 ▸). In the case of WAXS, by comparing equations (1) and (2), 

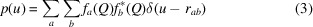

can be calculated as atomic scattering factor weighted PDDFs, where δ() is the Dirac delta function. Each PDDF is a histogram between chemical species for which the real part of 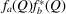 , 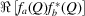 , is a common factor (Gelisio *et al.*, 2010 ▸). For computational time optimization, equation (3) becomes 

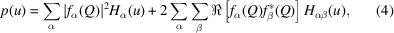

where α and β run over the number of chemical species in the NP, 

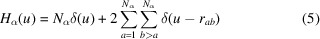

is the histogram of distances between atoms *a* and *b* of the same chemical species, and 

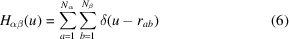

is the histogram of atomic distances where atoms *a* and *b* belong to the sets of *N*_α_ and *N*_β_ atoms of the different chemical species α and β, respectively.

For NPs of a given size and shape, simulated total scattering patterns of monodisperse systems embracing both SAXS and WAXS regions are obtained by the scattering power *P*(*Q*), equation (2), after computing the above PDDFs in equations (5) and (6) (Gelisio *et al.*, 2010 ▸; Cervellino *et al.*, 2015 ▸).

### 2.2. Polydisperse systems

In systems of non-identical monocrystalline NPs scattering independently from each other, that is dilute systems where no spatial correlation exists between the NPs (Zemb & Lindner, 2002 ▸) and secondary scattering processes are negligible (Morelhão & Cardoso, 1991 ▸; Valério *et al.*, 2020 ▸), 

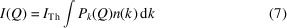

describes the scattered X-ray intensities, where *n*(*k*)d*k* stands for the number of NPs sharing a common property with value in the range from *k* to *k* + d*k*. The scattering power *P*_*k*_(*Q*) of the NPs in this range follows from equation (2) as long as they form a complete set of randomly oriented NPs. Here, the variable *k* refers to the size of the NPs, implying the assumption that they all have the same shape. For instance, *k* stands for the edge length *L* in systems of cubic NPs or the diameter *D* for spherical NPs.

According to the kinematical theory of X-ray scattering, all atoms of a given system of particles are subjected to the same incident wave, and the scattered waves thus produced suffer no further interaction with the system (Warren, 1990 ▸; Guinier, 1994 ▸). Under this kinematical approach, the integrated intensity 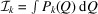 around each scattering peak in the WAXS region, referred to as a Bragg or diffraction peak, is proportional to the NP volume; that is 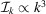 . On the other hand, while the peak area increases with the NP volume, the peak width 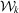 gets narrower inversely with the NP size *k*; that is 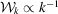 . Consequently, the maximum peak height at the Bragg position *Q*_0_ follows *P*_*k*_(*Q*_0_) ∝ *k* ^4^. There is also the scattering peak around the direct beam at *Q* = 0, the SAXS peak. In this case, equation (1) provides the squared number of scattering electrons or the squared volume (Guinier & Fournet, 1955 ▸; Guinier, 1994 ▸). Hence, this scattering peak maximum height follows *P*_*k*_(0) ∝ *k* ^6^.

In samples with a particle-size-distribution (PSD) function *n*(*k*), the resulting full width at half-maximum (FWHM), or simply the peak width 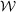 , follows from equation (7) as 

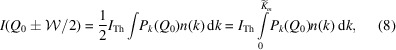

clearly establishing that 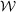 is related to the median value 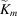 of the PSD function weighted by *P*_*k*_(*Q*_0_). Hence, 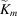 is the median value of the *k* ^*m*^-weighted PSD, that is

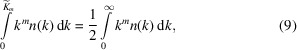

where *m* = 4 for all Bragg peaks and *m* = 6 for the SAXS peak.

In *Q* space, the widely known Scherrer equation (SE) is 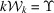 in the case of monodisperse systems with X-ray patterns, as given by *P*_*k*_(*Q*), equation (2). The NPs’ shape defines the exact value of the SE constant ϒ, and it may differ from one peak to another in the case of non-spherical NPs. It is always slightly different for the SAXS peak, even for spherical NPs. In the case of polydisperse systems with a general PSD, the same SE constants apply (Leoni, 2019 ▸; Morelhão & Kycia, 2022 ▸). A measure of the peak width 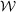 leads to the median value 

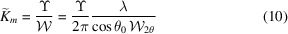

when estimating the NP size in the sample via the SE. Equation (10) also shows the most used form of the SE where 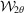 is the peak width as a function of the scattering angle 2θ, given that 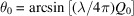 is the Bragg angle and is zero for the SAXS peak. The ϒ/2π ratio is commonly called the shape factor of the SE, whose value is close to 1; that is ϒ ≃ 2π. In this work, the ϒ values will be extracted from the monodisperse X-ray patterns of virtual NPs, since their values provide the size results from simulated patterns of polydisperse sizes. It is also a computational demonstration to verify theoretical values in the literature (Zemb & Lindner, 2002 ▸; Langford & Wilson, 1978 ▸; Scardi & Leoni, 2001 ▸) in the absence of samples of perfectly monodisperse sizes.

Before demonstrating that the SE leads to the medians of the PSD’s *m*th moment integrals, as stated in equation (10), the physical meaning of the SE in samples with PSD was unclear (Jensen *et al.*, 2006 ▸; Bergeret & Gallezot, 2008 ▸). What has previously been demonstrated is that the volume-weighted average size is obtained by taking the ratio of the total area under the peak divided by the peak intensity at maximum, also known as a measure of the integral breadth (Stokes & Wilson, 1942 ▸). After clarifying the meaning of the SE whether in the SAXS or WAXS regimes, a methodology for simultaneously evaluating dispersivity and crystallinity becomes feasible, as detailed hereinafter.

## 3. Materials and methods

### 3.1. Virtual nanoparticles

Vibrational disorder was introduced in the virtual NPs by adding [d*x*, d*y*, d*z*] = δ*r*[ζ_1_, ζ_2_, ζ_3_] to the mean atomic positions 〈**r**_*a*_〉 = [*X*_*a*_, *Y*_*a*_, *Z*_*a*_] as computed for perfectly periodic lattices. The random numbers ζ_*n*_ are in the range [0, 1], and the atomic disorder parameter δ*r* produces a Gaussian broadening of width δ*r* in the histograms of pair distances, corresponding to a nearly isotropic root mean square (RMS) displacement 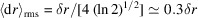 (Section S1). For each virtual NP, the histograms in equations (5) and (6) were calculated with a bin of width Δ*u* = 0.002 Å, as in 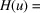 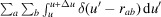 . Computer codes were written in C++ for parallel-processing mode with 36 cores. The execution environment was a virtual machine running a Debian-amd64 8.11 operating system (56 cores and 16 GB memory) hosted by an HPE ProLiant DL360 Gen10 Server with Xen kernel, with 64 cores of an Intel Xeon Gold 5218 CPU @ 2.30 GHz/256 GB memory. For an NP with *N*_at_ atoms of the same chemical species, the observed time to compute *H*_α_(*u*) [equation (5)] is 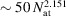 ps. For instance, an NP with *N*_at_ = 19 × 10^6^, having *N*_at_(*N*_at_ − 1) = 361 × 10^12^ pair distances, takes a processing time of 63 h. The atomic scattering factors *f*(*Q*) = *f*_0_(*Q*) + *f*′(λ) + *if*′′(λ) in equation (4) were calculated by routines asfQ.m and fpfpp.m, both available from Morelhão (2016 ▸). The integral in equation (2) leads to the scattering power of the NPs, as it becomes a discrete sum of the *p*(*u*) values on each histogram bin weighted by the corresponding 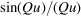 factor, that is 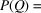 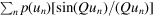 where *u*_*n*+1_ = *u*_*n*_ + Δ*u*.

### 3.2. Ceria nanocubes

CeO_2_ NPs were synthesized via a hydrothermal process by adaptation of a well established protocol (Mai *et al.*, 2005 ▸). First, two concentrations of 6 *M* (a sample labeled B5) and 12 *M* (samples labeled B11 and C1) sodium hydroxide (NaOH, P.A-A.C.S. 100%, Synth) were dissolved in 35 ml of deionized water. Next, 0.1 *M* cerium nitrate [Ce(NO_3_)_3_·6H_2_O, ≥99.9%, Sigma–Aldrich] precursor was dissolved in 10 ml of deionized water and added to the NaOH solution with stirring. For sample C1, 0.025 *M* urea (CH_4_N_2_O, P.A-A.C.S. 100%, Synth) was added as a structure-directing agent. After being stirred for 15 min, the slurry solution was transferred into a 100 ml stainless steel vessel autoclave, heated at 180°C in an electric oven for 24 h and allowed to cool at room temperature. The final product was collected by centrifugation, followed by washing in deionized water and ethanol five times each. Finally, the precipitate was dried in an electric oven at 80°C for 12 h.

### 3.3. Electron microscopy analysis

Scanning electron microscopy (SEM) images were obtained with JEOL microscopes operating at 5 kV. Samples were prepared by drop-casting aqueous suspensions over double-stick carbon tape, drying under ambient conditions and sputtering a 3 nm platinum coating to improve the signal–noise ratio.

### 3.4. X-ray diffraction analysis

XRD on CeO_2_ powder samples was performed in a D8 Discover (Bruker) with a LYNXEYE XE-T detector. We used Cu *K*α radiation (1.5418 Å), a Bragg–Brentano θ–θ goniometry and step-scanning mode: step size of 0.015° and counting time of 2 s per step. The sample spinning velocity was 15 r min^−1^. A NIST corundum 676a standard (Cline *et al.*, 2011 ▸) at room temperature provides the peak widths 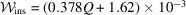 Å^−1^ used to account for the instrumental broadening of the diffractometer. Peak widths 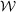 , without instrumental broadening, were obtained by using both Gaussian–Gaussian (GG) and Gaussian–Lorentzian (GL) deconvolution functions (Kielkopf, 1973 ▸; Olivero & Longbothum, 1977 ▸), 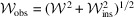 and 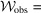 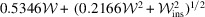 , respectively. 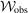 stands for the actual FWHM extracted by line-profile fitting with pseudo-Voigt functions. Alternatively, Rietveld refinement for crystallite-size analysis via the *GSAS-II* software (Toby & Von Dreele, 2013 ▸) is also provided (Section S2).

#### 3.4.1. SAXS analysis

SAXS data were acquired in a Xeuss 2.0 system (Xenocs, France) sourced by a microfocus GeniX3D X-ray generator, Cr *K*α radiation (2.2923 Å), with a FOX3D collimating mirror and ultra-high resolution mode where the beam cross section is set to 0.4 × 0.4 mm^2^. A Pilatus 300 K (Dectris, Switzerland) area detector was placed at 6.46 m from the sample. The spectral line width of the characteristic radiation limits the X-ray longitudinal coherence length to ∼310 nm. In the transverse direction, the coherence length is determined by X-ray collimation and estimated by the equipment manufacturer to approach 500 nm. The total acquisition time per sample was 1 h. The intensities scattered on the detector area were converted into curves as a function of *Q* by using the in-house software available at the Center of Multi-User SAXS Equipment of the Institute of Physics, University of São Paulo, Brazil, where the measurements were carried out. Background scattering from Kapton films, holding the powder samples in the vacuum chamber, has been subtracted from the intensity curves, and no beam-stopper shadow corrections were applied.

## 4. Computational results

**Figure 1:**
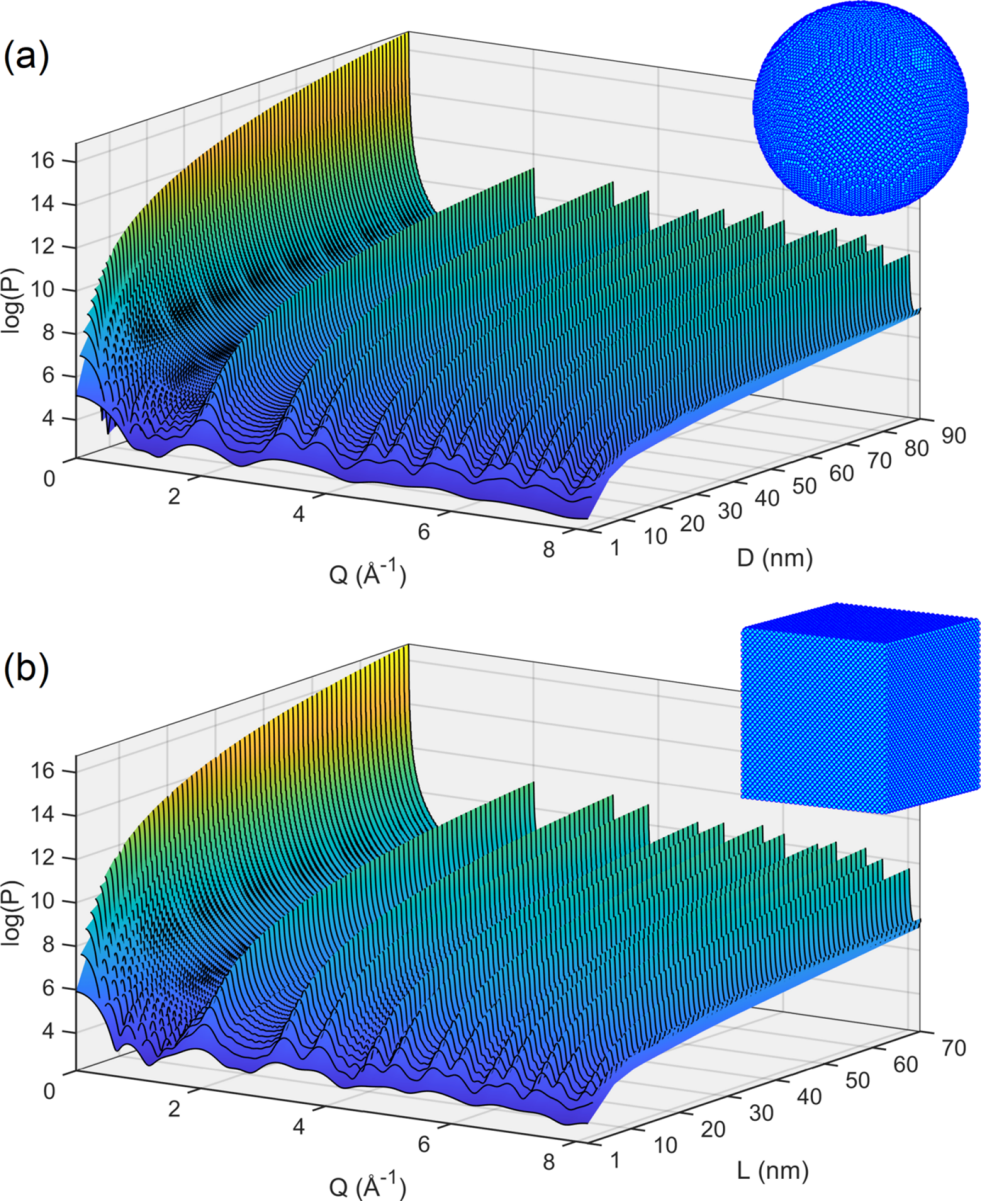
Scattering power *P*(*Q*), equation (2), obtained by PDDF calculation in virtual NPs of silicon. (*a*) Spherical NPs of diameter *D*, ranging from 1 to 90 nm, and (*b*) cubic NPs of edge *L*, ranging from 1 to 70 nm. An RMS displacement of 1.4 pm mimics vibrational disorder at all atomic sites in the NPs. The insets show examples of spherical (*D* = 20 nm) and cubic (*L* = 16 nm) virtual NPs with ∼200 000 atoms each.

Fig. 1 ▸ displays *P*(*Q*) curves of spherical and cubic silicon NPs representing X-ray patterns of monodisperse systems. Single-element NPs were considered to optimize computer time. The two sets of simulated patterns with sizes covering the broadest possible range serve three purposes: to verify values and uncertainties of the SE constants for crystallites of nanoscale sizes; to simulate size dispersion by composing weighted patterns based on the monodisperse patterns; and to evaluate at least two distinct NP shapes, in one of which the diffraction peak widths are susceptible to the crystallographic orientation of the NP facets. Weighted patterns and verified SE constants are needed to quantify SAXS and XRD sizes from polydisperse systems of virtual NPs.

**Figure 2:**
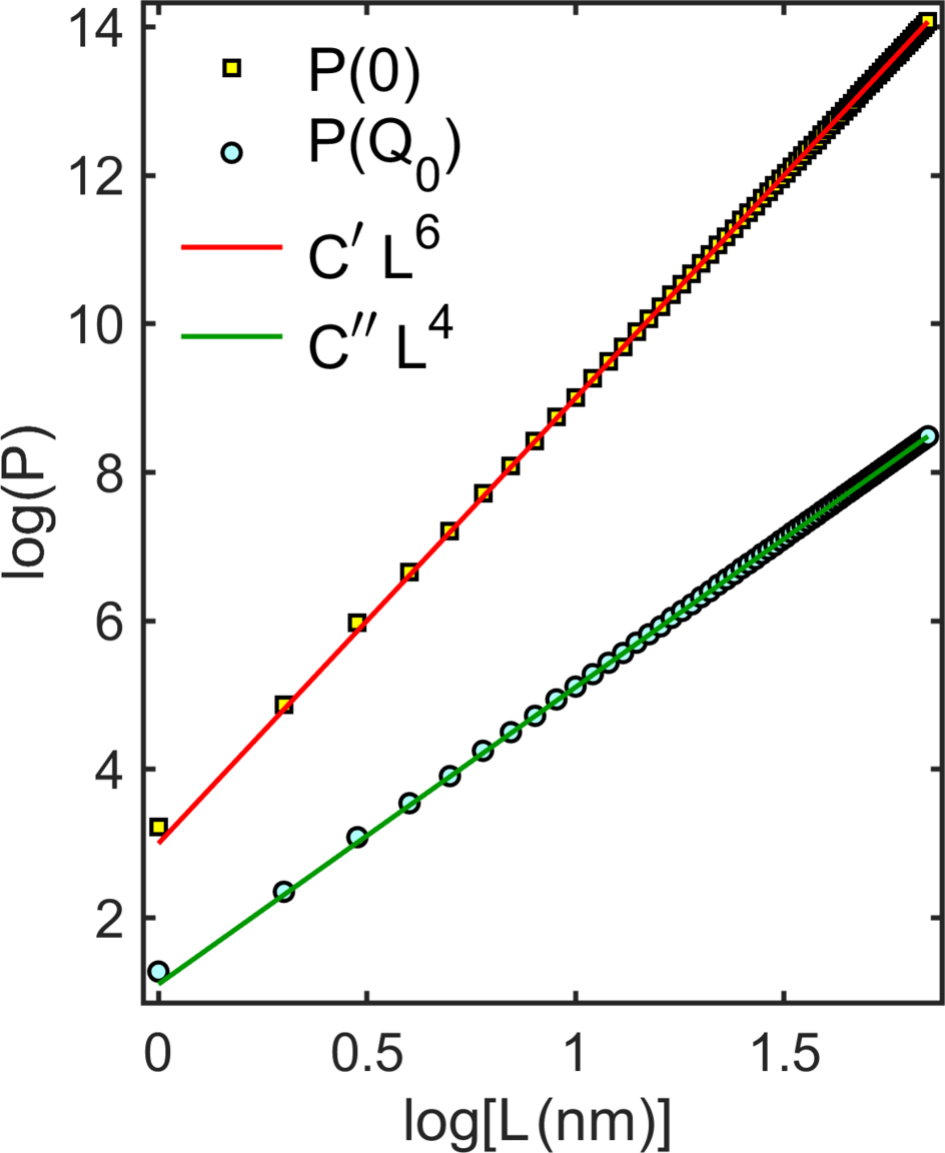
SAXS (squares) and WAXS (circles) peak maximum heights as a function of NP cube edge length *L*, as obtained from the *P*(*Q*) curves in Fig. 1 ▸(*b*) for *Q* = 0 and *Q*_0_ = 3.2723 Å^−1^, respectively. Expected behavior (solid lines) of the maxima according to the X-ray kinematical theory, that is *P*(0) = *C*′*L* ^6^ and *P*(*Q*_0_) = *C*′′*L* ^4^, is also shown; the proportionality constant adjusted values are *C*′ = 1003 and *C*′′ = 12.77.

Fig. 2 ▸ compares the maximum heights of the scattering peak *P*(0) and of one Bragg peak *P*(*Q*_0_ > 0) as a function of the cube edge *L*, showing that they are proportional to *L* ^6^ and *L* ^4^, respectively. Similar plots are obtained for spherical NPs as a function of the diameter. According to equation (9), knowing the exact behavior of peak height versus size is important because it establishes the proper moment integral of the PSD function.

**Figure 3:**
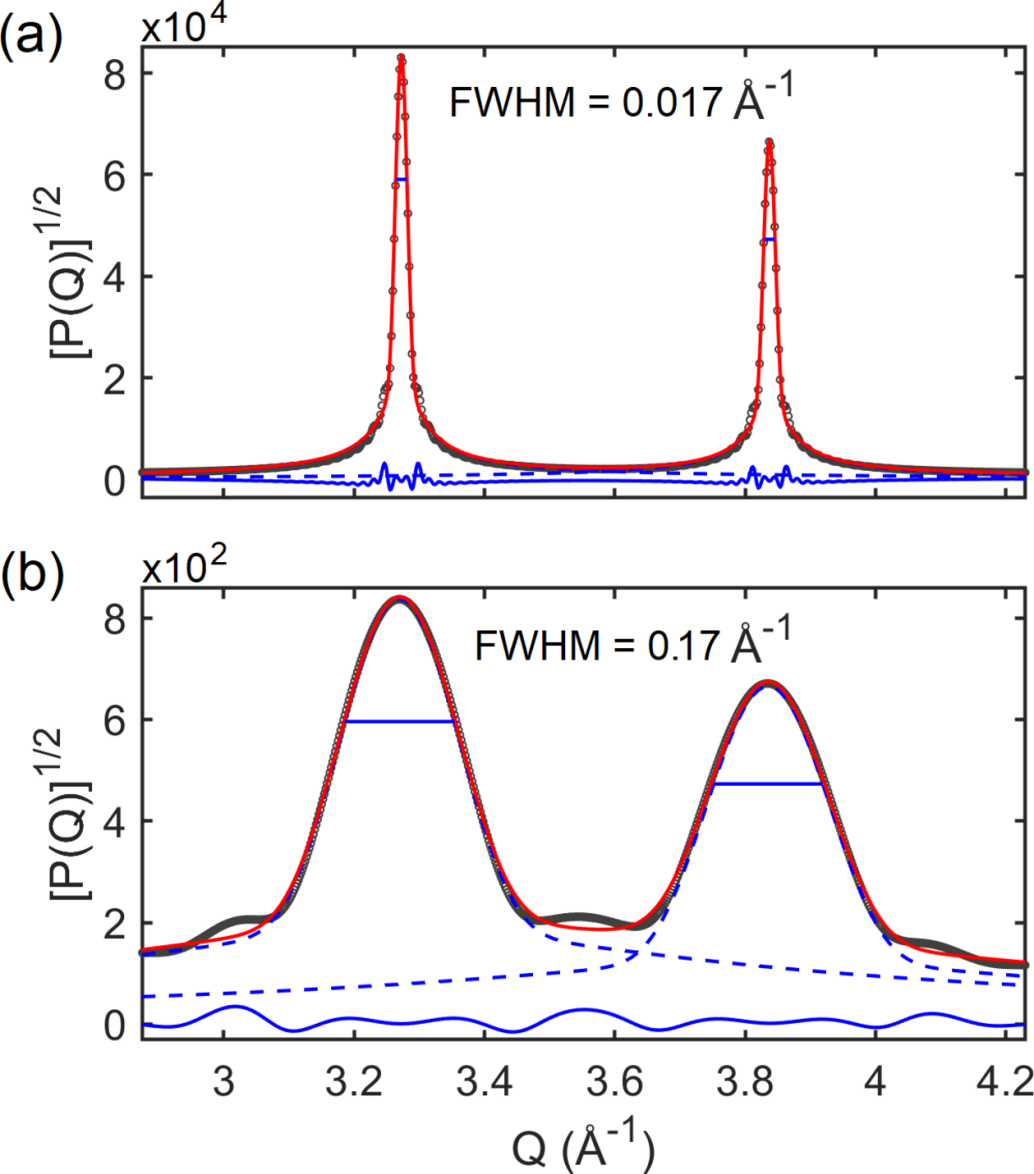
Nearby Bragg peaks of monodisperse NPs with two distinct diameters: (*a*) *D* = 40 nm and (*b*) *D* = 4 nm. They include simulated peaks (black open circles), line-profile fitting (red lines) with two pseudo-Voigt functions (blue dashed lines) and residue analysis (blue line). A factor of 10 in diameter ratio leads to factors of 10^−1^ in width and 10^4^ in height of the peaks.

**Figure 4:**
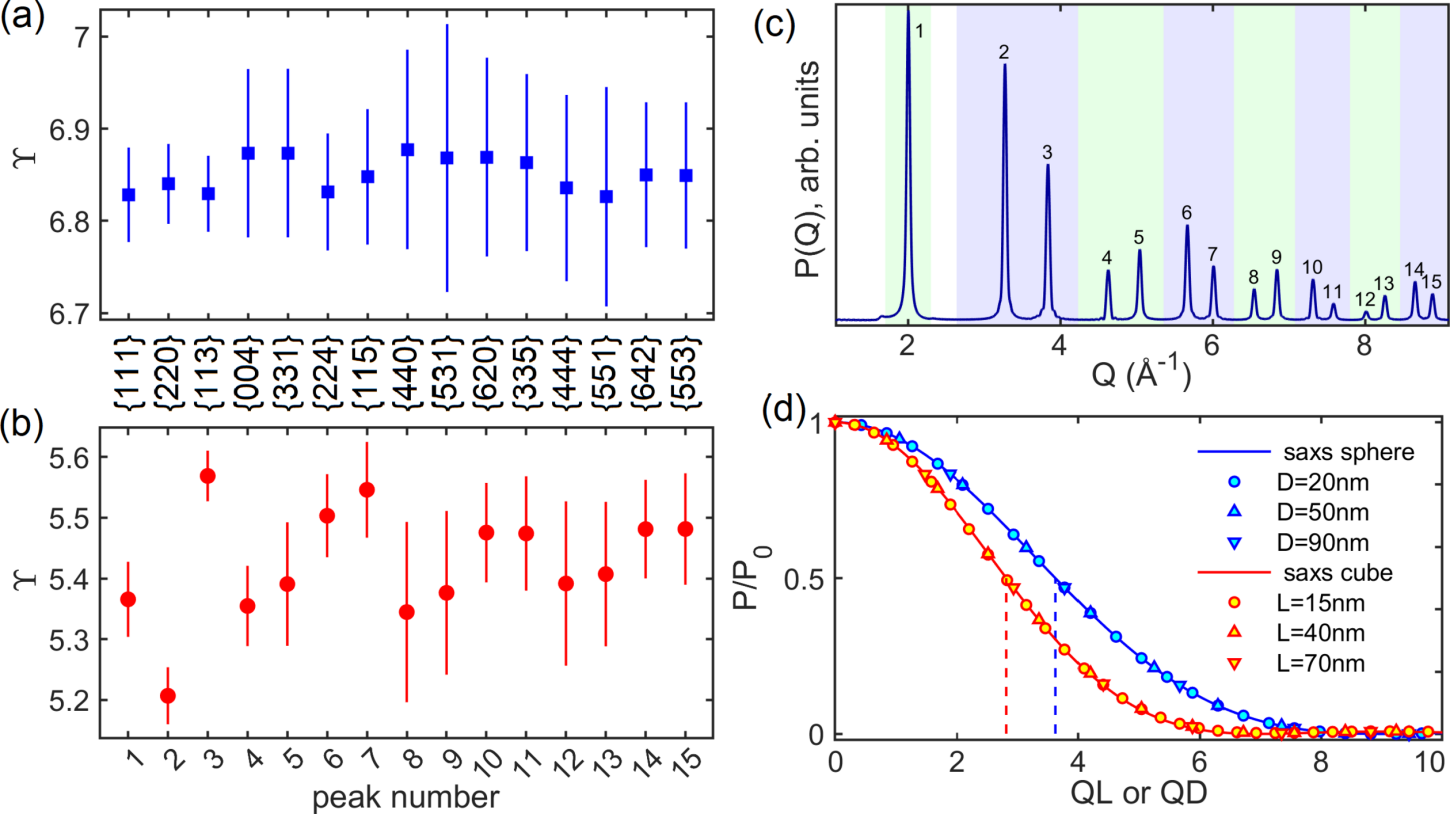
(*a*), (*b*) SE constant ϒ as obtained from the monodisperse patterns in Fig. 1 ▸ for (*a*) spherical and (*b*) cubic NPs with (001) facets. Vertical bars are the standard-deviation values computed for NPs with sizes varying from 3 to 90 nm. (*c*) Monodisperse pattern for NPs of edge *L* = 10 nm, showing numerical identifiers and intervals of fitting (shaded areas of different colors) of the XRD peaks used to determine the SE constants. The Bragg reflection family indices are indicated between the (*a*) and (*b*) panels. (*d*) SAXS curves of a few NP sizes (symbols) plotted against *QD* or *QL* and compared with the line-profile functions (solid lines) for SAXS intensity curves, equation (11). The half-height (dashed lines) for spheres and cubes occurs at *QD* = 3.630 and *QL* = 2.822, respectively.

For NPs smaller than 4 nm, Bragg peak overlapping is too severe, as can be seen in Fig. 1 ▸ for *D* or *L* smaller than 4 nm. Above this size, the peak widths were obtained by line-profile fitting. Examples of peak fitting are given in Fig. 3 ▸ for spherical NPs of diameter *D*, further confirming that Bragg peak intensities scale up with *D* ^4^ while peak width goes with *D* ^−1^. Figs. 4 ▸(*a*) and 4 ▸(*b*) summarize the mean ϒ values obtained by fitting the first 15 Bragg peaks of the *P*(*Q*) curves, while Fig. 4 ▸(*c*) indicates the intervals of fitting considered for each peak.

For spherical and cubic NPs, the obtained SE constants are ϒ_*D*_ = 6.84 ± 0.05 and ϒ_*L*_ = 5.4 ± 0.2, implying diffraction-based size determination through the SE with uncertainties of ∼1% and 4%, respectively. These are the minimum uncertainties, as measuring peak widths also contributes to some inaccuracy. The error bar of  ±0.2 in ϒ_*L*_ considers all variations in peak width due to the crystallographic orientation of NP facets, which is applicable to size estimation of cubic NPs without concern for the facet planes.

The SE constant ϒ_0_ for the SAXS peak differs from the SE constants for the diffraction peaks. However, its value is more accurate (uncertainty below 0.1%) than the diffraction ones since no nearby peak-overlapping effects compromise the evaluation. The term ϒ_0*D*_ = 7.260 for spheres and the term ϒ_0*L*_ = 5.643 for cubes, as shown in Fig. 4 ▸(*d*), where the computed *P*(*Q*) curves of the NPs at low *Q* are compared with line-profile functions of half widths ϒ_0*D*_/2*D* and ϒ_0*L*_/2*L*. Both line-profile functions have the general form

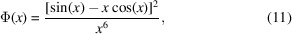

where 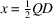 for spheres of diameter *D* (Guinier & Fournet, 1955 ▸) and 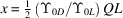 for cubes of edge *L*. Spheres and cubes have the same volume when *D*/*L* = 1.241, a close value to the obtained ratio ϒ_0*D*_/ϒ_0*L*_ = 1.286. Table 1 ▸ displays the SE constants for SAXS and XRD size analysis.

X-ray patterns of polydisperse samples carry distinct information about the size distribution. As SAXS and XRD intensity maxima have heights proportional to the NP sizes raised to different powers, 6 and 4, respectively, peak-width measurements provide the medians of the PSD’s sixth- and fourth-moment integrals, as indicated in equation (9). Computational demonstration of these direct correlations between peak width and median values takes the monodisperse patterns, Fig. 1 ▸, weighted by a chosen PSD function as a way to simulate the size-dispersivity effect.

**Figure 5:**
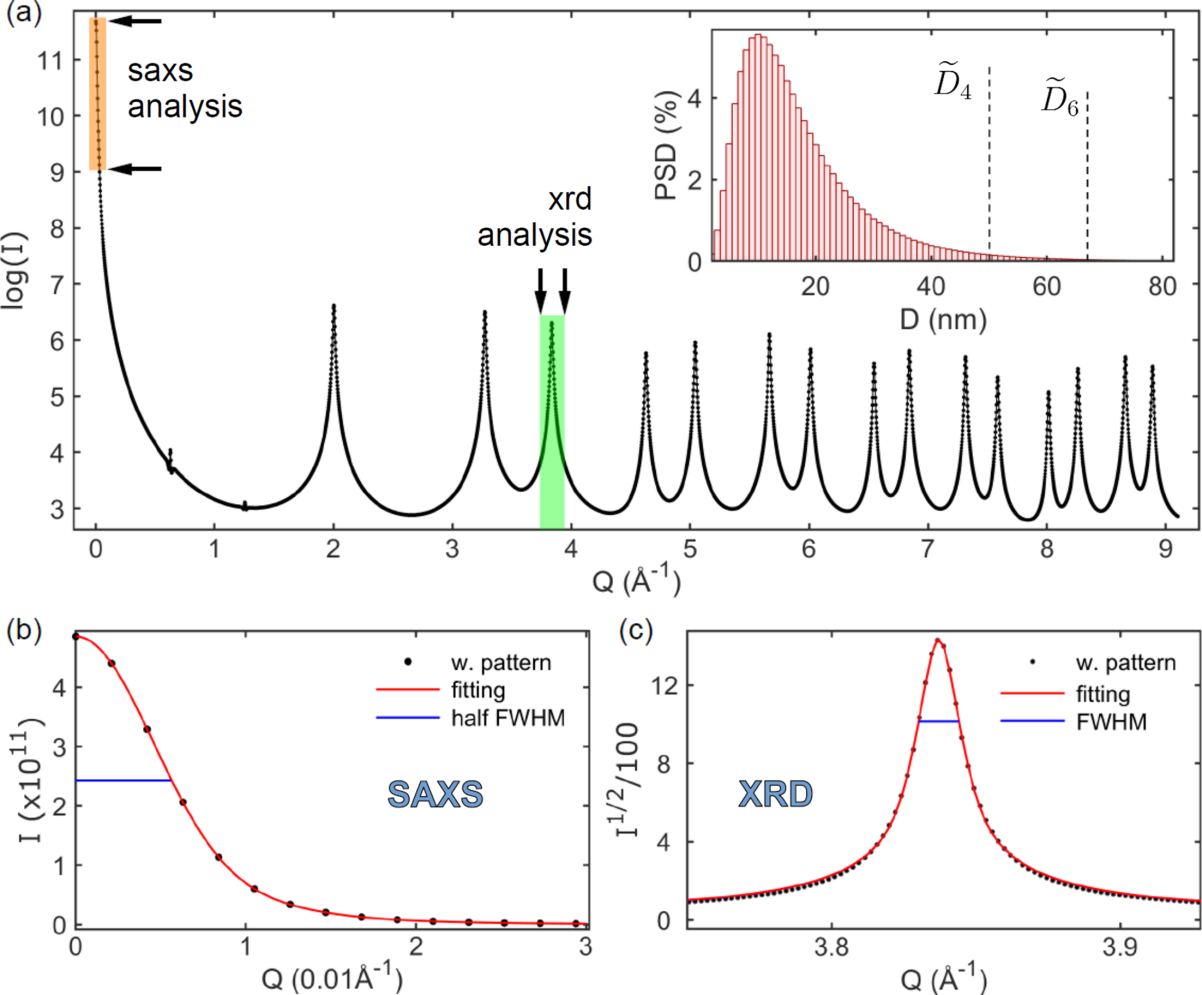
(*a*) Simulated X-ray pattern (dots with line) of polydisperse spheres given in number of scattering electrons, that is *I*(*Q*) in equation (7) with *I*_Th_ = 1. The inset shows a weighting function for the monodisperse patterns in Fig. 1 ▸(*a*) taken as a PSD function with diameter median values 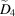 and 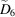 . Shaded areas and arrows indicate intervals in *Q* for SAXS and XRD size analysis. (*b*) SAXS peak of the weighted pattern (dots) and corresponding curve fitting (solid red line) based on the analytical expression of the SAXS intensity curve for spheres, equation (11). (*c*) Bragg peak of the weighted pattern (dots) and line-profile fitting (solid red line) based on the pseudo-Voigt function. Heights at half maximum of the scattering and diffraction peaks are indicated (horizontal blue lines). Peak widths (FWHM): 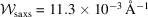 and 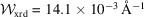 .

For example, Fig. 5 ▸(*a*) presents the simulated pattern of a polydisperse system of spheres with a discrete lognormal PSD function of 1 nm size increment in the range from 1 to 90 nm. The corresponding diameter median values for the PSD moment integrals are 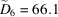 nm and 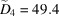 nm, Fig. 5 ▸(*a*) (inset). Both values are much larger than the 10 nm value of the PSD mode. Figs. 5 ▸(*b*) and 5 ▸(*c*) show the SAXS and XRD analysis of the simulated pattern, respectively. The SAXS peak of half-width 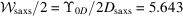 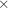 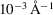 provides the SAXS size result of *D*_saxs_ = 64.3 nm, while the Bragg peak of width 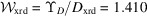 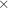 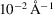 provides the XRD size result of *D*_xrd_ = 48.5 nm. Within the 1 nm accuracy of this numerical demonstration, the obtained size results perfectly agree with the corresponding median values of the PSD moment integrals. Table 2 ▸ shows median values and size results for narrower PSDs from the simulated patterns. Besides the excellent agreement between these values, Table 2 ▸ (last column) also reports that the Gaussian fraction *x* in the pseudo-Voigt line profile decreases as the size dispersion increases. The larger the size dispersion, the more Lorentzian-like the XRD peaks. Despite changes in the line-profile aspect, the SE constants for monodisperse systems work for polydisperse sizes, emphasizing the relationship between peak widths and median values of the weighted PSDs.

**Figure 6:**
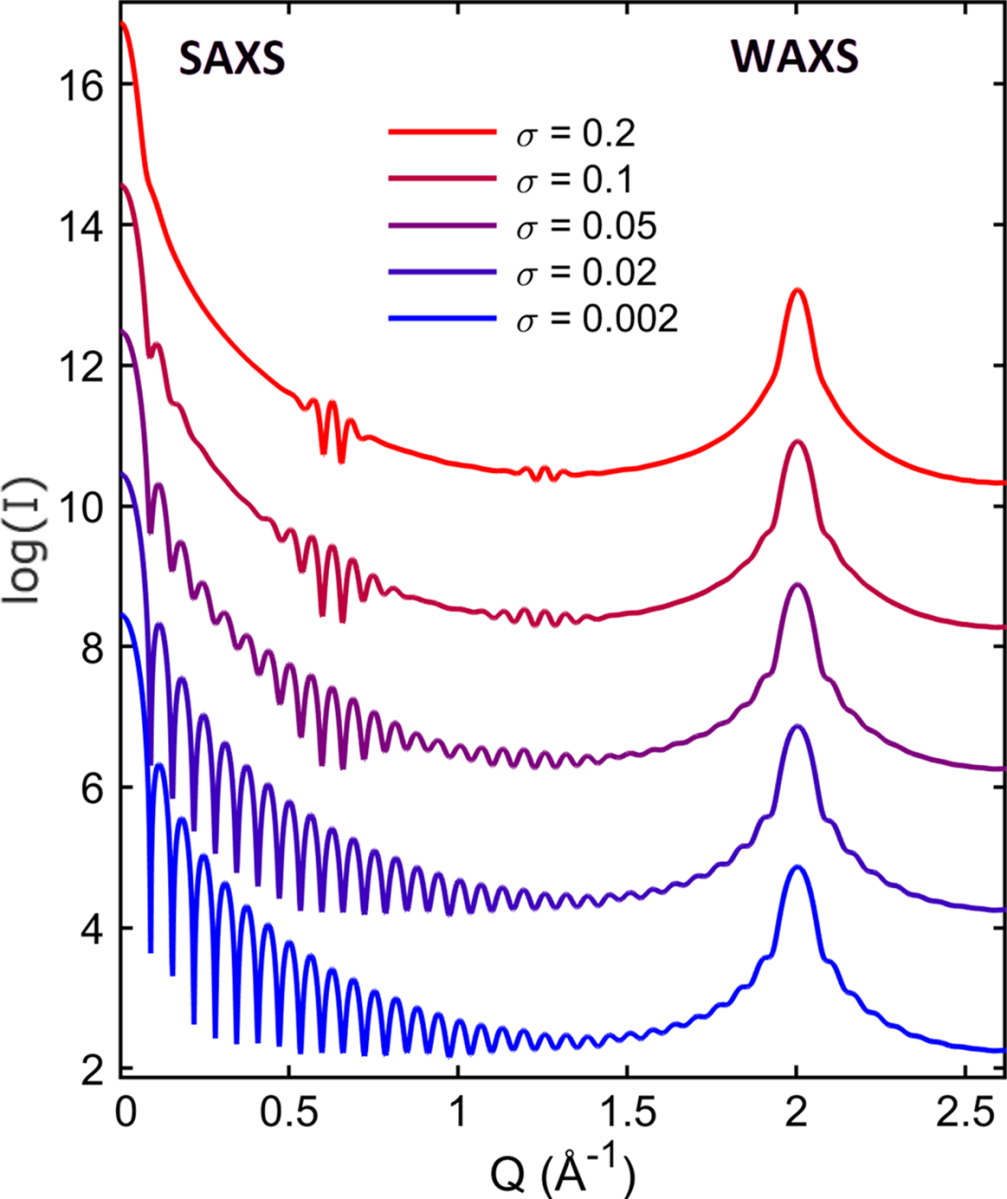
Simulated X-ray patterns of polydisperse cubes given in number of scattering electrons, that is *I*(*Q*) in equation (7) with *I*_Th_ = 1. The patterns cover the SAXS region and the first diffraction peak in the WAXS region. Lognormal functions of 10 nm mode and standard deviations of σ ≤ 0.2 were used for weighting the monodisperse patterns in Fig. 1 ▸(*b*).

Another difference between SAXS and XRD with respect to size distribution is the much higher susceptibility of the SAXS fringes when compared with the smooth ripples around the diffraction peaks in the WAXS region, as shown in Fig. 6 ▸. In cases where the size results from median values are practically the same, as in Table 2 ▸ (first and second rows), SAXS fringes can easily grade size distributions with lognormal standard deviations narrower than σ = 0.2. However, even for highly monodisperse NPs, as shown by the SAXS fringes, the question of how to quantify the degree of crystallinity still remains. An approach to address this issue is presented later in this work.

## 5. Experimental results

**Figure 7:**
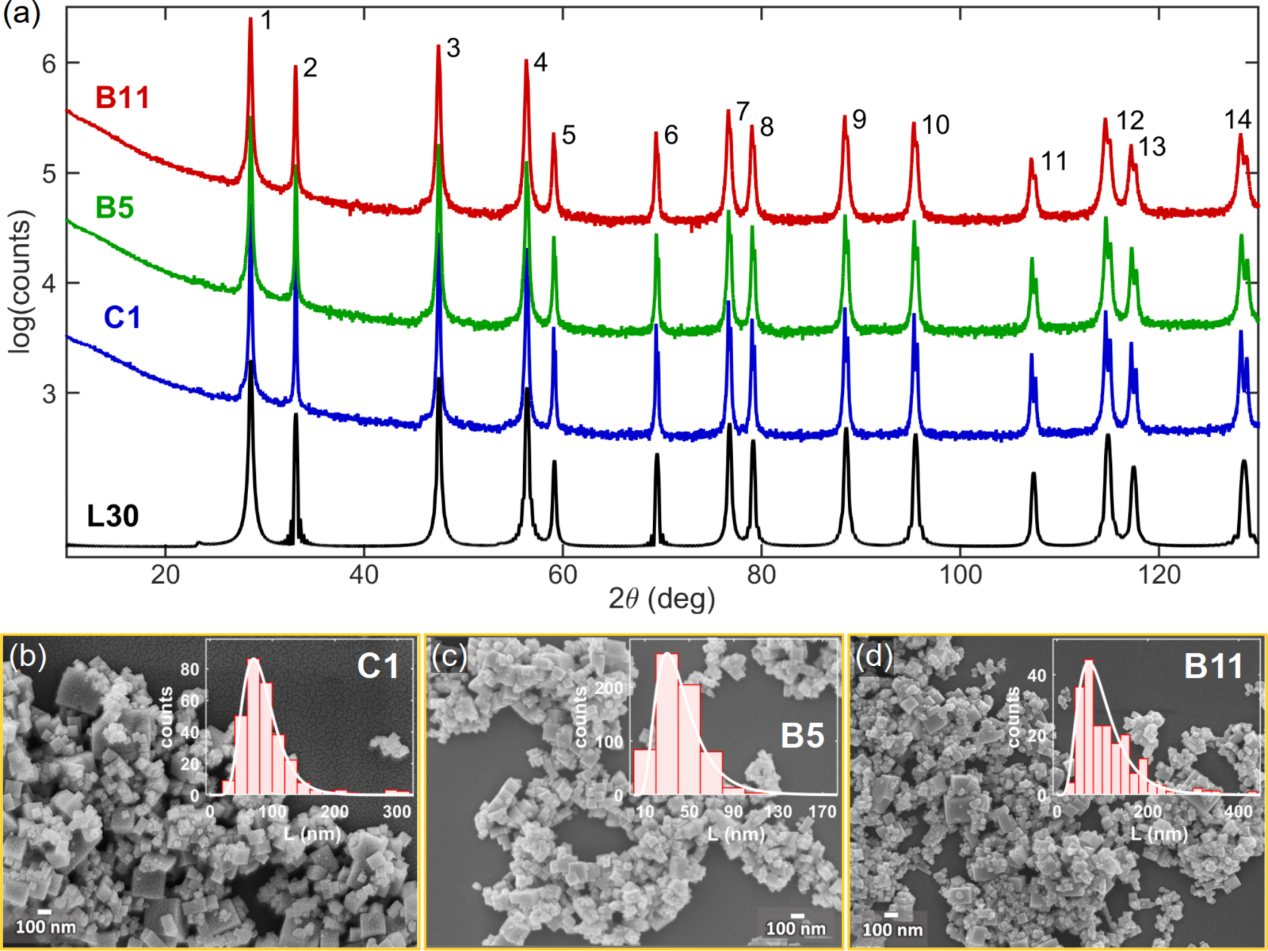
(*a*) XRD patterns of powder samples C1, B5 and B11 of CeO_2_ NPs with Cu *K*α radiation. A simulated pattern (L30) for a monodisperse system of cubic NPs of edge *L* = 30 nm is also shown as a reference, Cu *K*α_1_ only, and 〈d*r*〉_rms_ = 1.4 pm in the virtual NP is used to compute the Ce–Ce, Ce–O and O–O PDDFs in equations (5) and (6). (*b*), (*c*) SEM images and histograms (insets) of apparent size distribution for each powder sample as indicated.

**Figure 8:**
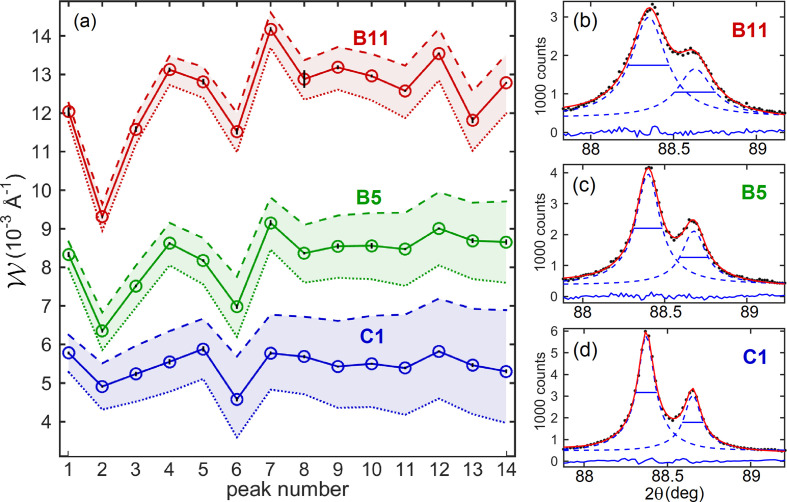
(*a*) Experimental peak width 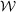 (open circles connected by lines) from XRD line-profile analysis in CeO_2_ cubic NPs, samples B11, B5 and C1, as obtained after the GG deconvolution of the measured FWHM values (dashed lines). Vertical error bars represent the statistical fluctuation of the fitting procedure. Peak widths after the GL deconvolution (dotted lines) are also shown. (*b*)–(*d*) Examples of line-profile fitting (solid red lines) of one doublet peak (scattered black dots) with pseudo-Voigt functions (dashed blue lines); see Section S2 for the other peaks as well as for the Rietveld analysis.

Fig. 7 ▸ shows XRD patterns and SEM images of the CeO_2_ powder samples. According to the images, all samples have cubic-like NPs with sizes ranging from about ten to hundreds of nanometres, Figs. 7 ▸(*b*)–7 ▸(*d*). The apparent size distributions highly depend on the ensemble of NPs in each image. On the other hand, the XRD patterns reveal differences in the median values of particle sizes from one sample to another. This is seen qualitatively by the greater or lesser overlap of the *K*α radiation doublet peaks at higher angles, as in peaks 12 to 14 in Fig. 7 ▸(*a*). See also the zooms of peak 9 in Figs. 8 ▸(*b*)–8 ▸(*d*), indicating that the largest crystalline NPs or crystallites are present in sample C1 and the smallest ones are present in sample B11. Quantitative results of peak widths are shown in Fig. 8 ▸(*a*), where the peak widths differ little from one reflection to another but the differences between samples are evident. The average widths after GG deconvolution, 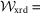 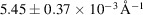 (sample C1), 8.25 ± 0.78 × 10^−3^ Å^−1^ (sample B5) and 12.5 ± 1.2 × 10^−3^ Å^−1^ (sample B11), are used for size measurements with standard deviations as the uncertainty. The SE constant for cubic NPs, ϒ_*L*_ = 5.4 ± 0.2, leads to the XRD size results, 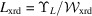 , in Table 3 ▸ (column 2). The NPs were considered strain free as the peak widths show no systematic variation as a function of *Q*.

**Figure 9:**
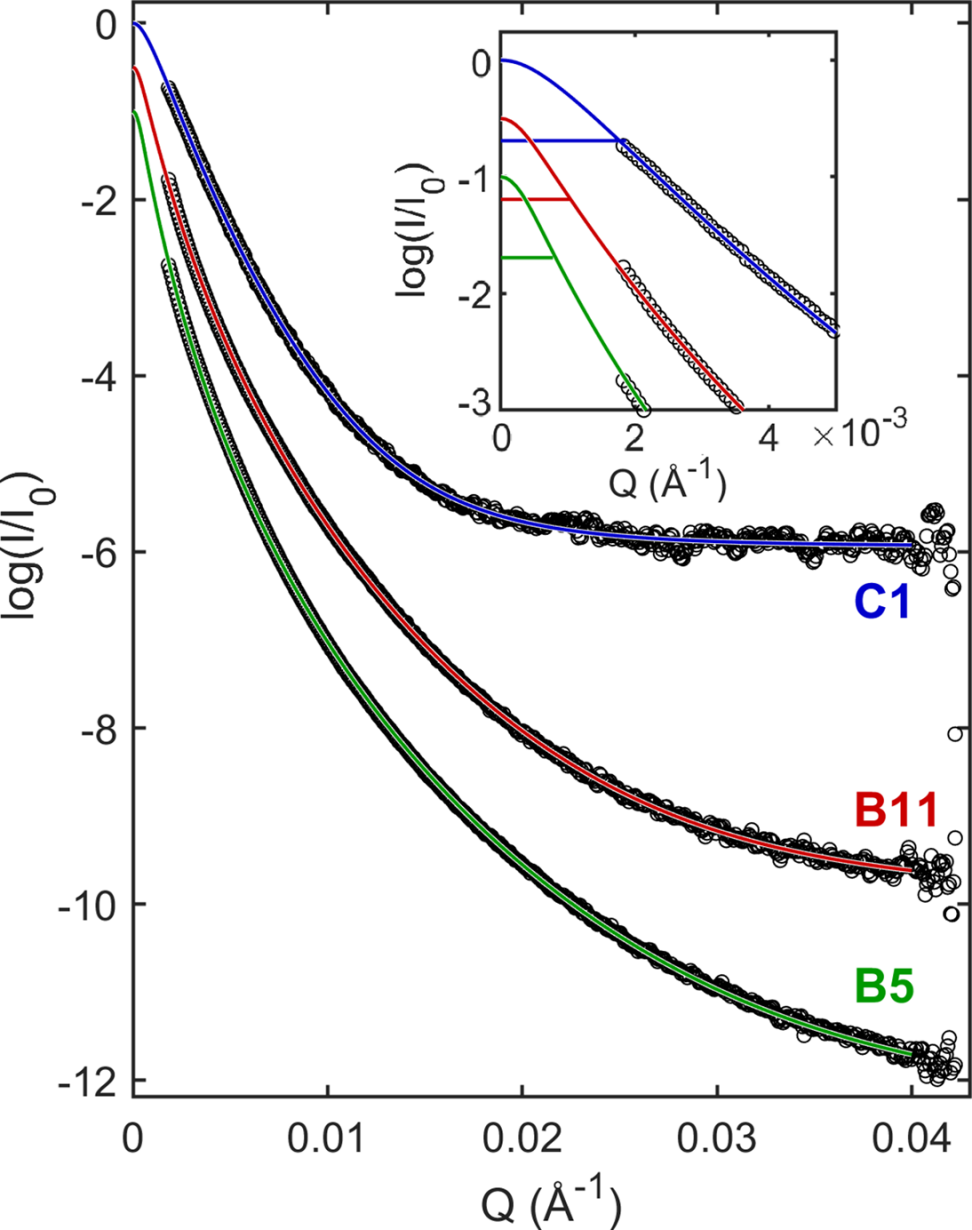
SAXS intensity curves, experimental (open circles) and adjusted (solid lines), from CeO_2_ cubic NPs, powder samples B5, B11 and C1. *I*/*I*_0_ stands for normalized intensity. Adjusted curves were obtained by the *Y*(*Q*)-fitting function in equation (14). Ultra-small *Q* regions are highlighted in the inset, where the height at half-maximum of each adjusted curve is indicated (horizontal solid lines).

Fig. 9 ▸ shows the SAXS measurements of samples C1, B11 and B5, as well as the best-fit curves of the data. As the intensity at *Q* = 0 is inaccessible in the used SAXS equipment, the SAXS peak half-widths 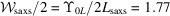 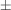 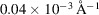 (sample C1), 1.04 ± 0.02 × 10^−3^ Å^−1^ (sam­ple B11) and 0.82 ± 0.03 × 10^−3^ Å^−1^ (sample B5) were estimated by extrapolating the fitted curves to *Q* = 0 (inset of Fig. 9 ▸). The uncertainties were estimated by repeating the fitting procedure several times after adding extra statistical noise to the data. The SE constant for cubic NPs, ϒ_0*L*_ = 5.64, leads to the SAXS size results, 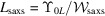 , in Table 3 ▸ (column 3).

## 6. Discussion

To interpret as adequately as possible the discrepant XRD and SAXS size results, such as *L*_xrd_ and *L*_saxs_ in Table 3 ▸, it is necessary first to evaluate possible discrepancies for actual size distributions as if there is no interaction between NPs and their sizes have exactly the coherent lengths of their crystal lattices, that is considering a dilute system of perfect crystalline NPs. This evaluation requires choosing an appropriate PSD function. In the particular case of one of the most common probability functions for size distribution, which is the lognormal function (Kril & Birringer, 1998 ▸; Kiss *et al.*, 1999 ▸; Jensen *et al.*, 2006 ▸), the *m*th moment integral has analytical solution 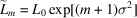 for its median value given in terms of the most probable size (mode) *L*_0_ and standard deviation σ (Section S3). By taking the medians 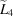 and 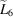 as the XRD and SAXS size results, respectively, the corresponding size-distribution parameters follow from 

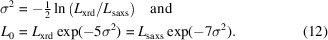

This shows that the discrepancy 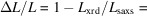 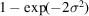 in size results arises as an exclusive consequence of the size-dispersion parameter σ under the idealized conditions mentioned above. The contribution of partial crystallization to the size discrepancy is taken into account here within the assumption that the volume fraction ξ between the crystalline domain and the whole NP volume is nearly constant concerning the NP size. Then, the size discrepancy accounting for both effects becomes 

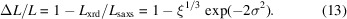

In samples with narrow size distributions and crystalline NPs, as represented by simulated patterns with σ ≤ 0.2 in Table 2 ▸ (first and second rows), no detectable discrepancy is introduced as long as the error bars in the XRD and SAXS size values are more significant than 5%. In other words, compatible size results, Δ*L*/*L* < 0.08 [from equation (13) with ξ = 1 and σ = 0.2], only occur for systems of crystalline NPs with narrow size distributions. For example, SAXS curves displaying well defined fringes are typical signatures of monodisperse sizes (Zemb & Lindner, 2002 ▸; Wu *et al.*, 2018 ▸). In such cases, measuring *L*_xrd_ ≃ *L*_saxs_ proves that the NPs are also highly crystalline, ξ > 0.78 [from equation (13) with Δ*L*/*L* < 0.08 and σ = 0].

Equation (12) imposes no upper limit on the dispersion parameter σ. However, lognormal functions with σ > 0.8 represent distributions with a broad size dispersion ranging from a few nanometres to near a micrometre. By stipulating this σ value as the upper limit of physically acceptable size dispersivity when synthesizing NPs, discrepancies where Δ*L*/*L* > 0.72 are strong evidence that other effects contribute to the observed discrepancy value. According to this criterion, only sample C1 shows a discrepancy (Δ*L*/*L* = 0.38) small enough to be totally explained in terms of size distribution. When this is the case, the parameters σ and *L*_0_ of the distribution can be directly obtained from equation (12), as presented in Table 3 ▸ (columns 5 and 6). However, the Δ*L*/*L* ≥ 0.81 size discrepancy for samples B5 and B11 is enormous, suggesting that SAXS has measured NP sizes significantly larger than their crystal lattices.

From the SAXS perspective, the size-distribution parameters were already determined when fitting the SAXS curves. The analytical expression in equation (11) adapted for cubes of edge *L*, weighted by *L* ^6^ and by a lognormal function *n*(*L*), led to a parametric SAXS fitting function of

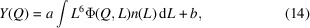

with four adjustable parameters: normalization factor *a*, constant background intensity *b*, and the lognormal parameters *L*_0_ and σ. The data-fitting quality relies on a genetic algorithm that minimizes the mean-squared error of the log-transformed data (Wormington *et al.*, 1999 ▸). The best fits of the SAXS data are those shown in Fig. 9 ▸, while the *L*_0_ and σ values characterizing the size distributions from the SAXS perspective are given in Table 3 ▸ (columns 7 and 8). The median values 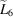 of these distributions match the *L*_saxs_ values of the half-width measurements. Without XRD measurements, all available information about the samples’ size dispersivity has to rely on the fitting procedures of the SAXS curves in Fig. 9 ▸. As the fitting quality is very good for the analyzed samples, there is no room to question the reliability of the SAXS results or the methodology used based on polydisperse sizes of non-interacting cubic NPs. The scenario is entirely different when results from another bulk technique, such as XRD, are introduced. For comparison purposes, the σ and *L*_0_ size-distribution parameters from fitting the SAXS curves allow us to calculate the expected XRD size results, 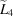 (Table 3 ▸, column 9).

**Figure 10:**
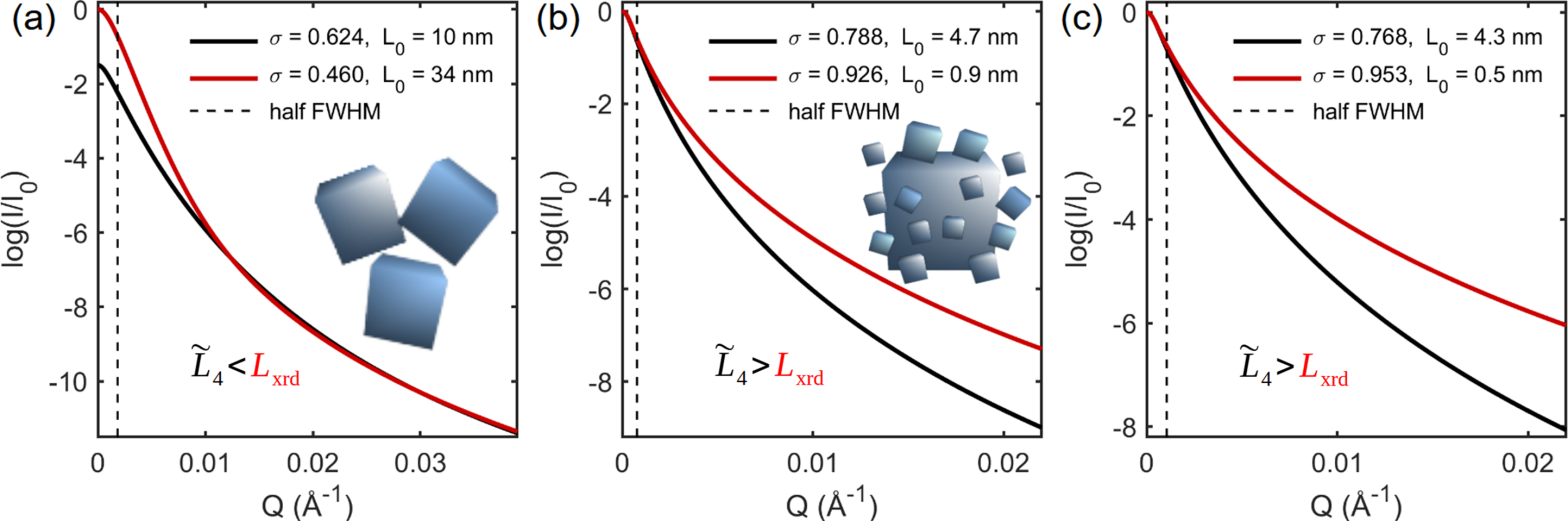
SAXS curves simulated for polydisperse systems of non-interacting cubic NPs with lognormal size-distribution parameters σ and *L*_0_ from Table 3 ▸, as indicated. In each panel, the curves have close values of *L*_saxs_ and 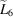 , but significantly different values of *L*_xrd_ and 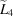 (Table 3 ▸). (*a*) Curves with different intensity maxima at *Q* = 0 and similar asymptotes, as caused by the NP–NP exclusion distance effect (inset). (*b*), (*c*) Curves with different asymptotes and similar intensities around *Q* = 0, as caused by an interface blurring effect due to very small particles (inset).

For sample C1, 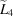 is smaller than *L*_xrd_, showing that the dispersion parameter σ = 0.623 optimized by curve fitting leads to the physical inconsistency of the NP crystalline domains being larger than the NPs themselves. This result illustrates the relevance of combining XRD and SAXS size results to evaluate the reliability of methodologies based on a single bulk technique. A possible cause of this inconsistency is an intensity reduction centered at *Q* = 0 [see Fig. 10 ▸(*a*)], characteristic of highly packed systems of NPs with similar sizes where the scattering becomes susceptible to the NP–NP exclusion distance (NP impenetrability) (Zemb & Lindner, 2002 ▸; Morelhão, 2016 ▸).

For samples B5 and B11, 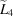 is larger than *L*_xrd_, indicating a physically feasible situation where SAXS probes NP sizes larger than their crystalline domains. In ideally dilute systems where the NP–NP distances are longer than the coherence length of the incident X-rays (*Materials and Methods* [Sec sec3]), the possible explanation goes towards NPs of partial crystallization. When that is the case, estimating the crystalline volume fraction 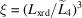 is possible within the assumptions leading to equation (13). Then, the volume fractions of crystalline material in the NPs of samples B5 and B11 are 24 ± 8% and 15 ± 5%, respectively. However, powder samples are generally far from dilute systems. For example, the NPs appear very clustered in the SEM images in Figs. 7 ▸(*c*) and 7 ▸(*d*). This allows us to consider other effects, such as samples with a broad size dispersion of crystalline NPs where many small particles wrap around the larger ones, blurring particle interfaces from the SAXS perspective and producing an apparent size effect of much larger particles [Fig. 10 ▸(*b*), inset]. Particle-interface properties are known to affect the SAXS curve asymptotic behavior, as in Porod’s law (Ciccariello *et al.*, 1988 ▸). The cause of 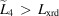 is the difference in asymptotic behavior [Figs. 10 ▸(*b*) and 10 ▸(*c*)], supporting the hypothesis that SAXS is seeing NP aggregates instead of individual NPs.

Proper interpretation of the XRD size result is a crucial point when comparing results from the two techniques, and it can be highlighted here by taking the results for sample B11 as an example. The *L*_xrd_ value accessed via XRD peak width through the SE, equation (10), is the median value 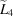 of the actual *L* ^4^-weighted size distribution. Although the median 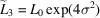 of the volume-weighted size distribution can be much closer to the actual XRD size result, its comparison with *L*_xrd_ leads to a misleading analysis for the following reasons. Sample B11 has 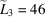 nm according to the SAXS results in Table 3 ▸ (columns 7 and 8), a value that perfectly matches the XRD size result of this sample, *L*_xrd_ = 43 ± 4 nm. This shows that, by misinterpreting the XRD size as 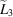 , size dispersion alone can explain the XRD and SAXS size discrepancy, leaving no room to question the SAXS fitting procedure, NP–NP interaction effects and NP crystallinity. In other words, the size discrepancy leads to a narrower size dispersion, and the sample appears more dilute and crystalline when *L*_xrd_ is mistaken for 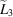 .

Concurrently with interpreting XRD size results correctly, there is also the need to establish the reliability of the obtained size values. Here two approaches have been considered. One approach was the line-profile fitting to the individual peaks with local background corrections rather than the whole pattern fitting to the diffraction data, widely known as Rietveld refinement. The former is feasible for small unit-cell materials like CeO_2_ and can lead to more accurate FWHM values as the fitting optimization is independent for each peak, as opposed to the fitting optimization for the whole pattern. The best fittings achieved by the two approaches are compared in Section S2. Fig. 8 ▸(*a*) shows the original FWHM values from individual fittings as well as the resulting peak widths after GG and GL deconvolutions. The most significant difference between these two deconvolutions, of ∼20% in size, is observed for sample C1: 99 ± 8 nm (GG) against 118 ± 9 nm (GL). The GL deconvolution is suitable when instrumental and size broadenings have previously been deconvolved from the standard-sample FWHMs, resulting in a smaller instrumental broadening to be deconvolved from the NP samples’ FWHMs. As the standard-sample FWHMs, that is the 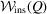 function, without previous deconvolution were considered for instrumental-broadening correction, the *L*_xrd_ sizes in Table 3 ▸ were obtained by using the GG deconvolution. These size values are in very good agreement with those obtained via Rietveld refinement (see Section S2, Table S1 of the supporting information).

By comparing chemical routes from the cerium nitrate precursor towards the synthesized samples B5 (NaOH 6 *M*), B11 (NaOH 12 *M*) and C1 (NaOH 12 *M* + CH_4_N_2_O 0.025 *M*), it is evident that sodium hydroxide acts as a nucleation agent while urea slows down nucleation and favors crystal growth. This eventually narrows the size distribution around larger sizes, as seen for sample C1. The relatively small size discrepancy (Δ*L*/*L* = 0.38) reported for this sample suggests highly crystalline NPs. In synthesizing samples B5 and B11, the higher the concentration of sodium hydroxide, the smaller the XRD size result, indicating its action mainly as a nucleation agent. Assertions about the actual size dispersivity and crystallinity of CeO_2_ NPs are compromised by the spontaneous self-aggregation observed in the series of samples analyzed here, making the preparation of dilute samples for proper SAXS measurements unfeasible. Reliable analytical procedures for further controlling size, size dispersivity and crystallinity are possible by comparing SAXS and XRD results in light of how these bulk techniques weight the size distribution. However, the feasibility of preparing dilute samples has to be also a concern when synthesizing the NPs. Besides dilute systems, it is also relevant to emphasize that ultra-SAXS instrumental configurations capable of actually measuring the scattering peak around the direct beam, like those configurations based on analyzer crystals (Chapman *et al.*, 1997 ▸; Pagot *et al.*, 2003 ▸; Antunes *et al.*, 2006 ▸; Rigon *et al.*, 2007 ▸; Morelhão *et al.*, 2010 ▸), improve the reliability of SAXS size results, as the peak maximum height and therefore its width are available measures irrespective of curve-fitting approaches. To evaluate size dispersivity and crystallinity of NPs in advanced X-ray instruments capable of accessing SAXS and WAXS simultaneously (Ilavsky *et al.*, 2018 ▸; Smith *et al.*, 2021*a* ▸; Shih *et al.*, 2022 ▸), the challenge is to prepare samples that are dilute enough for SAXS while sufficiently compact for Bragg peak signal analysis in the WAXS region.

**Figure 11:**
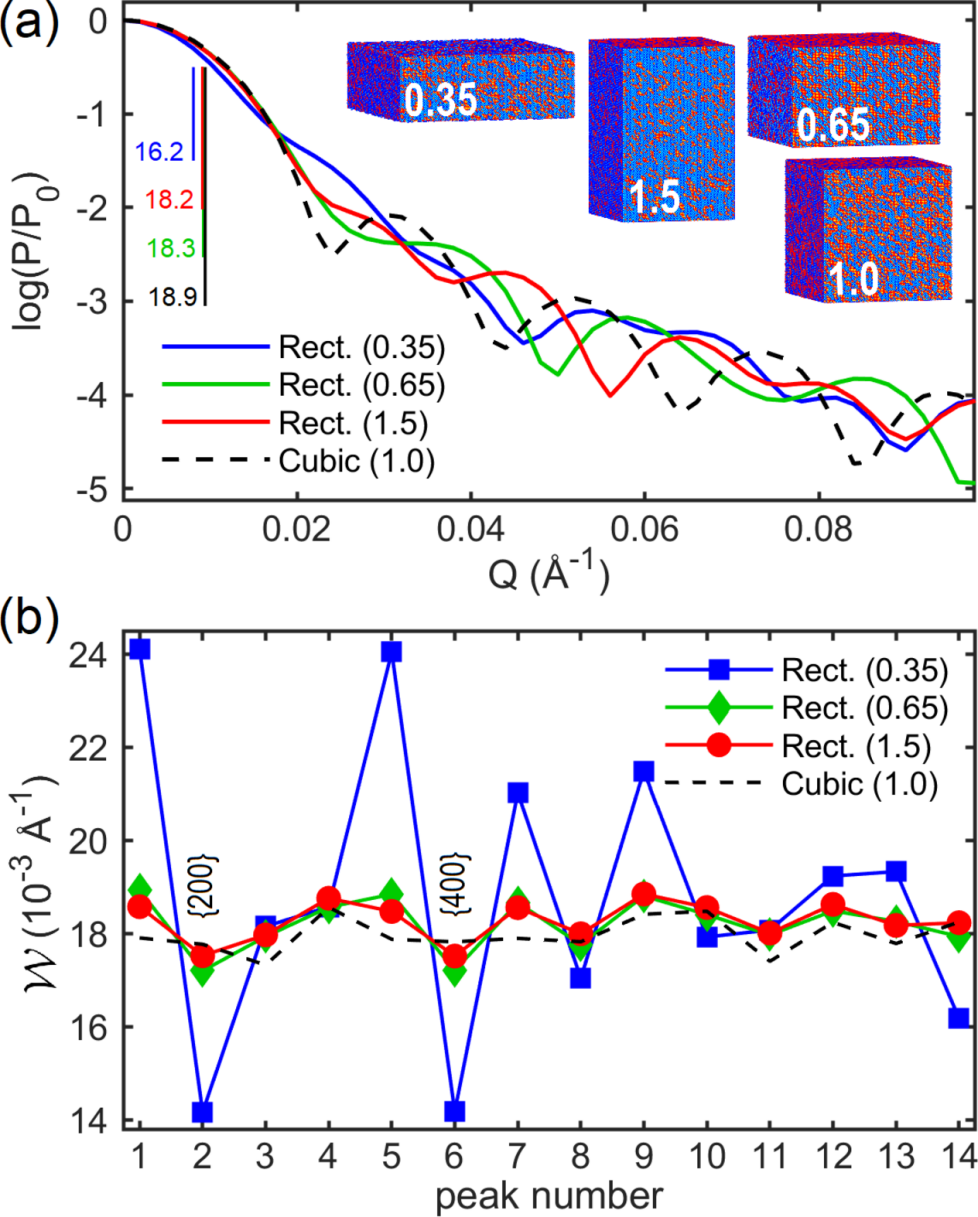
(*a*) SAXS curves for cubic-like NPs with rectangular shapes as given by the ratio of height/base-edge length (inset). FWHM values (vertical bars) are in units of 10^−3^ Å^−1^. (*b*) Bragg peak widths in monodisperse systems of CeO_2_ virtual NPs [inset, (*a*)]. All NPs have the same volume as a 30 nm cube. Peak numbers 2 and 6 stand for reflection families {200} and {400}, respectively. SAXS and WAXS simulation is done via equation (2). Peak widths were obtained by pseudo-Voigt line-profile fitting. NPs with 〈d*r*〉_rms_ = 1.4 pm were used for computing the Ce–Ce, Ce–O and O–O PDDFs [equations (5) and (6)].

In monodisperse systems, size information known as the radius of gyration is often obtained from the SAXS curve slope near *Q* = 0, above the half intensity and within the Guinier region (Guinier & Fournet, 1955 ▸). In polydisperse systems, the emphasis here was the behavior of the scattering peak FWHM as a function of NP size dispersion, as summarized by 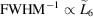 . However, going into a detailed discussion about the radius-of-gyration behavior with the size dispersion is beyond the scope of this work as it can be different from the 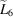 behavior (Morelhão & Kycia, 2022 ▸). The most intense part of the SAXS peak, including its FWHM, is susceptible most to the volume of the NPs, and hence to size dispersivity instead of shape dispersivity. For cubic-like NPs, this is demonstrated in Fig. 11 ▸(*a*), where the widths of the SAXS curves exhibit only negligible variations as a function of the rectangular-shape ratio, as given by height/base-edge length. Even for a significant deviation from the cubic shape, as for the NP with 0.35 ratio, there is only a relative variation of 14% in the curve FWHM value, which is very small in comparison with the size discrepancies reported in Table 3 ▸ (columns 2 and 3). The SAXS curve width provides the most reliable size information in general polydisperse systems.

Simulation of WAXS in systems of virtual NPs is a more appropriate approach to address shape dispersivity, as demonstrated in Fig. 11 ▸(*b*) where NPs with rectangular (100) facets have {200} and {400} Bragg peaks narrower than the others. This implies that the observation of {200} and {400} Bragg peaks with systematically narrower-than-average widths [peak numbers 2 and 6 in Fig. 8 ▸(*a*)] is consistent with the presence of a few NPs with pronounced rectangular shape, as seen in the SEM images in Figs. 7 ▸(*b*)–7 ▸(*d*). This observation also confirms that the peak-by-peak analysis of the X-ray patterns performed in this work has enough resolution to detect Bragg peak width variations related to deviation from the perfect cubic shape. Moreover, beyond the basic illustrative demonstrations in this work of the correlations between median values and peak widths, the role of polydispersity in X-ray scattering can be further investigated via simulation in virtual NPs as a relatively simple procedure. In general, theoretical approaches that have been deduced primarily on the basis of monodisperse systems, such as the asymptotic behavior of the SAXS curve as a function of *Q* (Guinier & Fournet, 1955 ▸; Pedersen, 1997 ▸; Zemb & Lindner, 2002 ▸; Li, 2013 ▸), can be tested in well controlled polydisperse systems instead of real systems where dispersivity properties are challenging to control.

## 7. Conclusions

Comparing SAXS and XRD size results creates possible procedures for attesting the reliability of both bulk techniques in accessing size-distribution parameters. In particular, in this work, SAXS intensity curves of ceria powders were precisely reproduced by fitting functions based on polydisperse sizes of non-interacting NPs. After introducing XRD size results, there was new information to question the reliability of the SAXS curve fitting. It revealed that NP–NP interaction effects in the SAXS curves compromise the assertive assessment of the NPs’ properties. Consequently, being able to prepare dilute samples for SAXS measurements is a preliminary requirement for analyzing the size dispersivity and crystallinity of NPs via the combined SAXS/WAXS methodology proposed here. The apparent crystallite size from the SE has to be compared with the median of the size distribution’s fourth-moment integral, where the size distribution here was obtained from SAXS curve fitting. In principle, it can also be obtained from other techniques, such as EM. Measurements of the SAXS curve width in sufficiently dilute samples lead to the median of the size distribution’s sixth-moment integral as the apparent size result. Discrepancy in apparent XRD and SAXS size results implies a relatively broad size distribution, one with lognormal standard deviation σ ≃ 0.2. Narrower size distributions (σ < 0.2) are shown by the SAXS fringes, while 80% is the highest detectable fraction of crystallized volume in the NPs when comparing apparent size results. The relationship provided in this work to correlate discrepancies of apparent sizes was based on a lognormal probability distribution function, valid for both particle and crystalline domain sizes. It is the first step towards more general situations that can be exploited by cross-analyzing SAXS and WAXS data.

## 8. Related literature

The following references are only cited in the supporting information for this article: Andrews (1997 ▸) and Glaisher (1871 ▸).

## Supplementary Material

Atomic disorder in virtual nanoparticles, line-profile analysis of X-ray diffraction Bragg peaks, and an analytical expression for median values of weighted lognormal functions. DOI: 10.1107/S1600576724003108/iu5050sup1.pdf

## Figures and Tables

**Table 1:** SE constants for SAXS (ϒ_0_) and XRD (ϒ) size analysis as determined from the monodisperse X-ray patterns in Fig. 1 ▸ XRD theoretical (theo.) SE constants are also shown.

NP shape	SAXS	XRD	Theo.
Sphere	ϒ_0*D*_ = 7.260 ± 0.002	ϒ_*D*_ = 6.84 ± 0.05	6.7525
Cube	ϒ_0*L*_ = 5.643 ± 0.002	ϒ_*L*_ = 5.4 ± 0.2	5.53 ± 0.14†

† Mean value and standard deviation for 52 main reflections in a cubic crystallite (Langford & Wilson, 1978 ▸).

**Table 2:** Median values 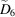 and 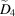 , with size results *D*_saxs_ and *D*_xrd_, as a function of σ, the PSD size-dispersion parameter in simulated X-ray patterns The numerical values, accurate to within 0.5 nm, are based on polydisperse patterns for spheres, *e.g.* Fig. 5 ▸(*a*). The PSDs are discrete 1 nm bin lognormal functions of standard deviation σ, 10 nm mode, with 90 nm as the maximum size. Parameter *x* stands for the Gaussian fraction in the pseudo-Voigt line profile of the XRD/Bragg peaks.

σ	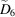 (nm)	*D*_saxs_ (nm)	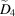 (nm)	*D*_xrd_ (nm)	*x*
0.1	10.3	10.8	10.1	10.6	0.93
0.2	12.8	13.4	11.7	12.2	0.84
0.4	30.1	31.8	21.8	22.6	0.69
0.6	66.1	64.3	49.4	48.6	0.25

**Table 3:** Size results *L*_xrd_ and *L*_saxs_, as determined by XRD and SAXS experiments in the C1, B11 and B5 powder samples, lognormal size-distribution parameters σ and *L*_0_ from equation (12) or by SAXS data fitting via equation (14), and median 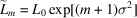 as the expected XRD (*m* = 4) and SAXS (*m* = 6) size results from the SAXS perspective only The last-digit uncertainties are in parentheses.

				From equation (12)		From equation (14)			
Sample	*L*_xrd_ (nm)	*L*_saxs_ (nm)	Δ*L*/*L*	σ	*L*_0_ (nm)	σ	*L*_0_ (nm)	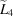 (nm)	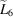 (nm)
C1	99 (8)	160 (4)	0.38	0.46 (8)	34 (9)	0.623 (4)	10.0 (2)	70 (3)	151 (6)
B5	66 (7)	345 (13)	0.81	0.93 (4)	0.9 (3)	0.788 (6)	4.7 (2)	105 (6)	364 (27)
B11	43 (4)	272 (6)	0.84	0.95 (4)	0.5 (1)	0.768 (3)	4.3 (1)	82 (3)	267 (10)
