# Supplementary material for: Implications of size dispersion on X-ray scattering of crystalline nanoparticles: CeO2 as a case study
Source: J Appl Crystallogr. 2024 May 31;57(Pt 3):793–807. doi: 10.1107/S1600576724003108 (PMC11151675; doi:10.1107/S1600576724003108)
Supplement: Supplementary file 1 [file j-57-00793-sup1.pdf]

JOURNAL OF APPLIED CRYSTALLOGRAPHY

Volume 57 (2024)

Supporting information for article:

Implications of size dispersion on X-ray scattering of crystalline nanoparticles: CeO_2 as a case study

Adriana Valério, Fabiane J. Trindade, Rafaela F. S. Penacchio, Bria C. Ramos, Sérgio Damasceno, Maurício B. Estradiote, Cristiane B. Rodella, André S. Ferlauto, Stefan W. Kycia and Sérgio L. Morelhão

1 ATOMIC DISORDER

The vibrational disorder has been treated within the frozen-in-time approach of the Debye scattering equation (DSE),[1–3] as it provides an exact calculation of the X-ray scattering at small and wide angles of randomly oriented nanoparticles (NPs). For materials made of a single element α, such as silicon NPs, the DSE is simply

$$P(Q) = |f_\alpha(Q)|^2 \int H_\alpha(u) \frac{\sin(Qu)}{Qu} du \tag{S1}$$

where $Q = (4\pi/\lambda)\sin\theta$ is the reciprocal vector modulus for a scattering angle 2θ and X-rays of wavelength λ. The pair distance distribution function (PDDF)

$$H_\alpha(u) = N_\alpha \delta(u) + 2\sum_{a=1}^{N_\alpha}\sum_{b>a}^{N_\alpha}\delta(u - r_{ab}) \tag{S2}$$

is the histogram of atomic distances $r_{ab} = |\boldsymbol{r}_b - \boldsymbol{r}_a|$ for NPs with N_α atoms; $\delta()$ stands for the Dirac delta function. The atomic scattering factor $f_\alpha(Q) = f_0(Q) + f'(\lambda) + if''(\lambda)$ in Eq. (S1) have been calculated by routines `asfQ.m` and `fpfpp.m`, both available at MatLabCodes(2016).[4] Small disorder in the atomic positions $\boldsymbol{r}_a = \langle \boldsymbol{r}_a \rangle + d\boldsymbol{r}$ were generated by adding $d\boldsymbol{r} = \delta r[\zeta_1, \zeta_2, \zeta_3]$ to the mean atomic positions $\langle \boldsymbol{r}_a \rangle = [X_a, Y_a, Z_a]$ of the crystal lattice. The random numbers ζ_n are in the range $[0, 1]$. The atomic disorder δr produces a broadening of width δr in the histograms of pair distances, as illustrated in Fig. S1 by disordering a single unit cell several times. A Gaussian of standard deviation

$$\sigma = \frac{\delta r}{2\sqrt{2\ln(2)}} = \sqrt{2\langle dr \rangle^2_{\mathrm{rms}}} \tag{S3}$$

fits very well this broadening, corresponding to an isotropic root-mean-square (RMS) displacement $\langle dr \rangle_{\mathrm{rms}} = \delta r/(4\sqrt{\ln 2}) \simeq 0.3\delta r$ around the mean atomic positions.

In NPs with sizes above a few nanometers, as the 4 nm diameter NP in Fig. S2, the PDDF obtained for a single δr-disordered NP is very similar to the average PDDF from an ensemble of NPs. The PDDFs are almost identical for the shortest distances, Fig. S2 (left panel). In contrast, the poor statistic from a single particle is more evident for the most extended distances, as seen in Fig. S2 (right panel). However, the NP scattering power $P(Q)$, that is, the X-ray scattering patterns from systems of randomly oriented NPs, exhibit tiny irrelevant differences only, as compared in Fig. S3. Bulk properties dominate for larger particles, and the poor statistic of the most extended distances is even more irrelevant. Therefore, for

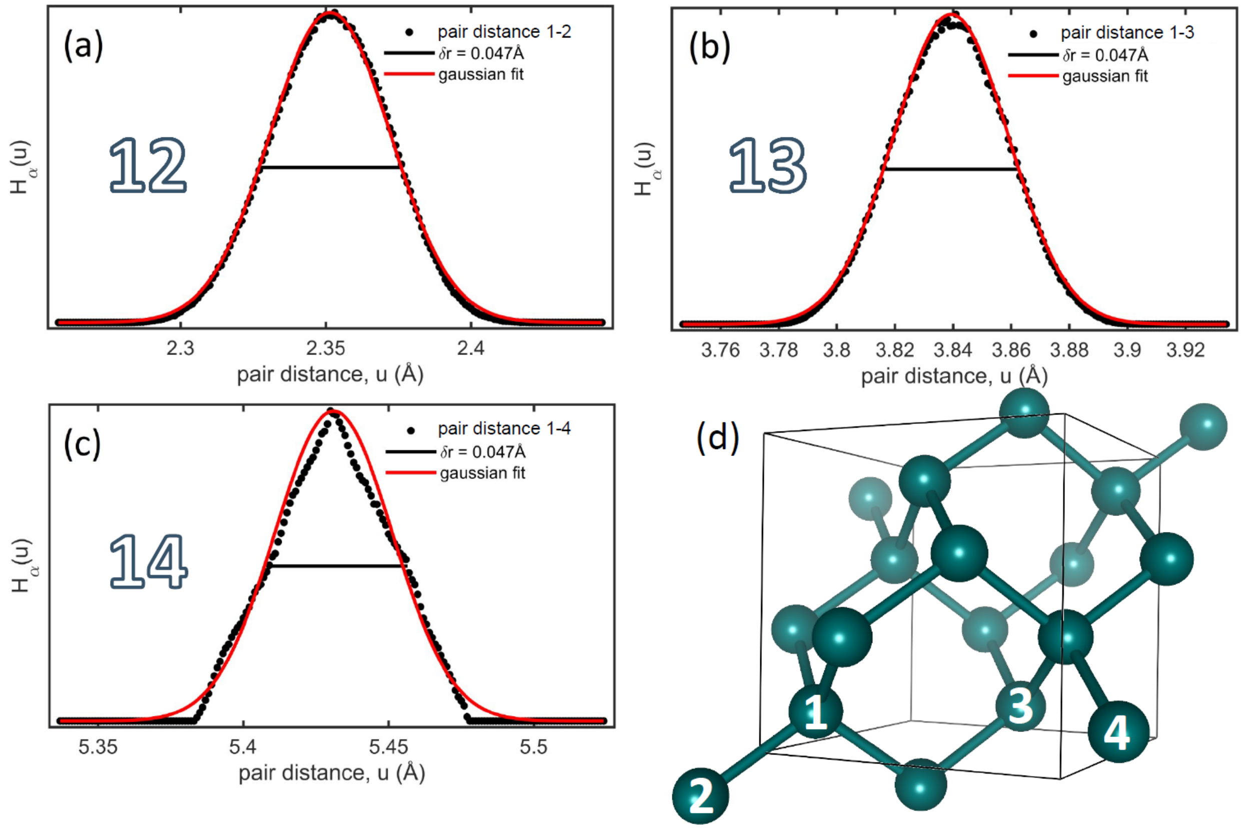

Figure S1: (a-c) Broadening in the histogram $H_\alpha(u)$ of pair distances caused by atomic vibration with RMS displacement $\langle dr \rangle_{\mathrm{rms}} = 0.3\delta r = 0.014\,\text{Å}$ in a silicon unit cell. The histograms (scatter dots) were computed with a bin of $0.001\,\text{Å}$ within an ensemble of 1000 unit cells with random disorder of 2%, that is $\delta r = 0.047\,\text{Å}$. Gaussian fits (solid red lines) with standard deviation $\sigma = 0.02\,\text{Å}$, Eq. (S3), are also shown. (d) Silicon unit cell. Atoms indicated by numbers were used to calculate the pair distances r_{12}, r_{13}, and r_{14} along the [111], [110], and [100] directions, respectively.

X-ray scattering pattern simulation, instead of concerning the average PDDF

$$\langle H_\alpha(u)\rangle = H_\alpha(u) * G(u) = N_\alpha\delta(u) + 2\sum_{a=1}^{N_\alpha}\sum_{b>a}^{N_\alpha} G(u - r_{ab}), \tag{S4}$$

given by the convolution of Eq. (S2) with a Gaussian function $G(u)$ of unit area and standard deviation σ, Eq. (S3), it is enough to compute the PDDF of a single δr-disordered NP.

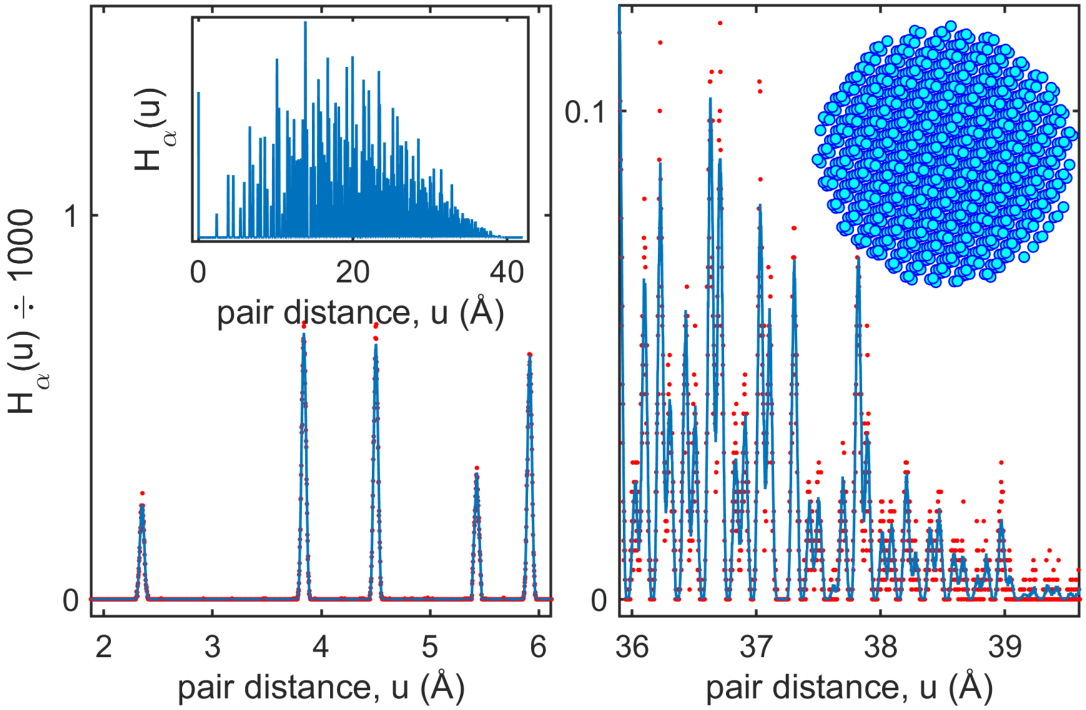

Figure S2: PDDF (red dots) of a 2%-disordered 4 nm diameter silicon NP compared to the average PDDF (solid blue line). Left panel: pair distances of the first neighbors. Right panel: longest distances in the NP. PDDF of the whole NP, as well as the NP itself are shown as insets. All distances are seen with a standard deviation $\sigma = 2$ pm. Total number of atoms in the NP: $H_\alpha(0) = 1672$ (inset, left panel). Bin width of 0.002 Å.

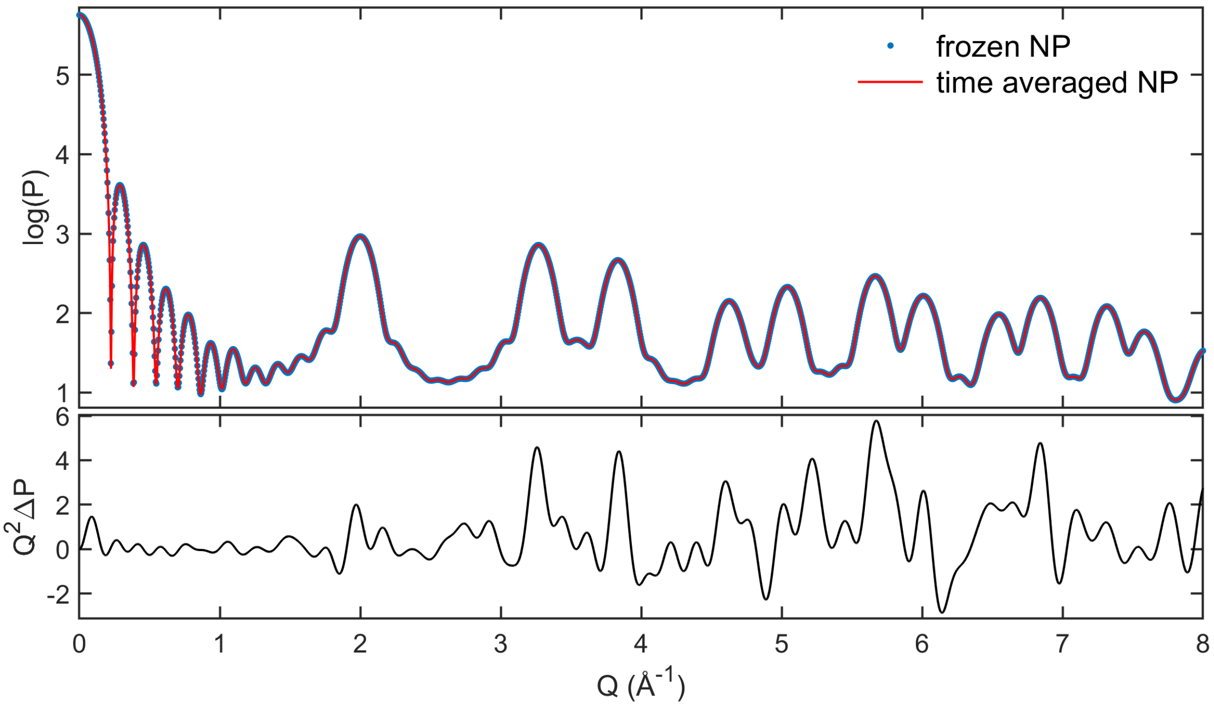

Figure S3: Top panel: Scattering power $P(Q)$, Eq. (S1), of a single 2%-disordered 4 nm diameter silicon NP (blue dots) in comparison to the average scattering from an ensemble of disordered NPs (solid red line). Bottom panel: Difference ΔP of the above curves multiplied by Q^2 to make visible on linear scale tiny differences in the wide-angle region ($Q > 1\,\text{Å}^{-1}$).

2 CRYSTALLITE SIZE ANALYSIS

Bragg peak widths, FWHM values, were measured by line profile fitting of the CuK_α radiation doublet peaks. It was carried out by a pseudo-Voigt double function

$$V(2\theta) = V_1(2\theta) + 0.485V_2(2\theta) \tag{S5}$$

where $V_n(2\theta) = x_n G_n(2\theta - 2\theta_n) + (1 - x_n)L_n(2\theta - 2\theta_n)$. G_n and L_n stand for Gaussian and Lorentzian functions, respectively, both of height h_n. θ_n is the Bragg angle for an atomic interplane distance d and walength λ_n ($\lambda_1 = 1.540562\,\text{Å}$ and $\lambda_2 = 1.544426\,\text{Å}$). For each hkl reflection family, there are 9 adjustable parameters: d, x_1, x_2, h_1, h_2, and the widths of the G_1, L_1, G_2, and L_2 functions. Background fitting is based on linear interpolation of the baseline intensity around the peaks, as detailed elsewhere.[5] Data fittings were driven by a genetic algorithm that minimizes the mean-square error of the data.[6]

Line profile fittings of individual Bragg peaks with the pseudo-Voigt double function $V(2\theta)$, Eq. (S5), for the C1, B5, and B11 samples are presented in Fig. S4, Fig. S5, and Fig. S6, respectively. In contrast, Fig. S7 shows the X-ray diffraction whole pattern fittings with the GSAS-II Python code.[7] The general relationship

$$\text{FWHM}(Q) = \frac{2\pi}{\lambda}\cos(\theta)\text{FWHM}(2\theta) \tag{S6}$$

have been used to convert between the FWHM values of Bragg peaks observed in intensity curves plotted either as a function of 2θ or Q. The $\mathcal{W}_{\text{obs}}$ values reported in the main text, Fig. 7, stand for FWHM(Q).

Instrumental peak broadening was determined from the X-ray pattern of a standard corundum powder in Fig. S7(a). Examples of line profile fitting of individual peaks with the $V(2\theta)$ function are shown in Fig. S8. The FWHM values, $\mathcal{W}_{\text{ins}}$, obtained from individual peak analysis are presented in Fig. S9(a) as a function of the scattering vector modulus Q.

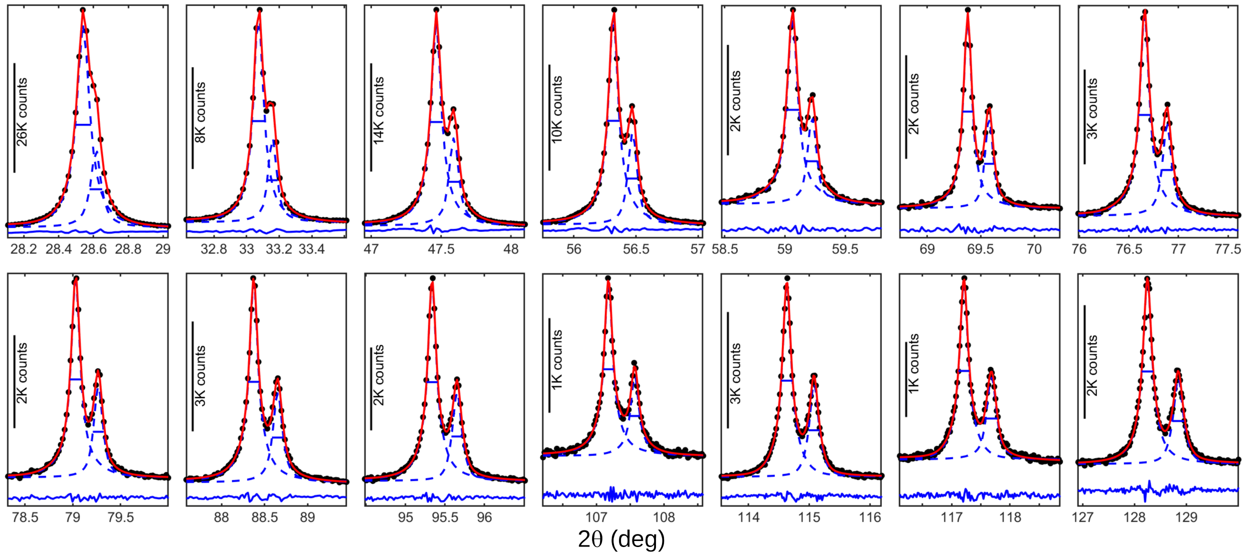

Figure S4: Sample C1. Line profile fitting (solid red lines) of $K_{\alpha 1}$ and $K_{\alpha 2}$ Bragg peaks (scatter dots) by using the pseudo-Voigt double function $V(2\theta)$, Eq. (S5). The FWHM of each $V_n(2\theta)$ function (dashed lines) are indicated by horizontal solid lines. The residuals of curve fitting are also shown (blue solid lines).

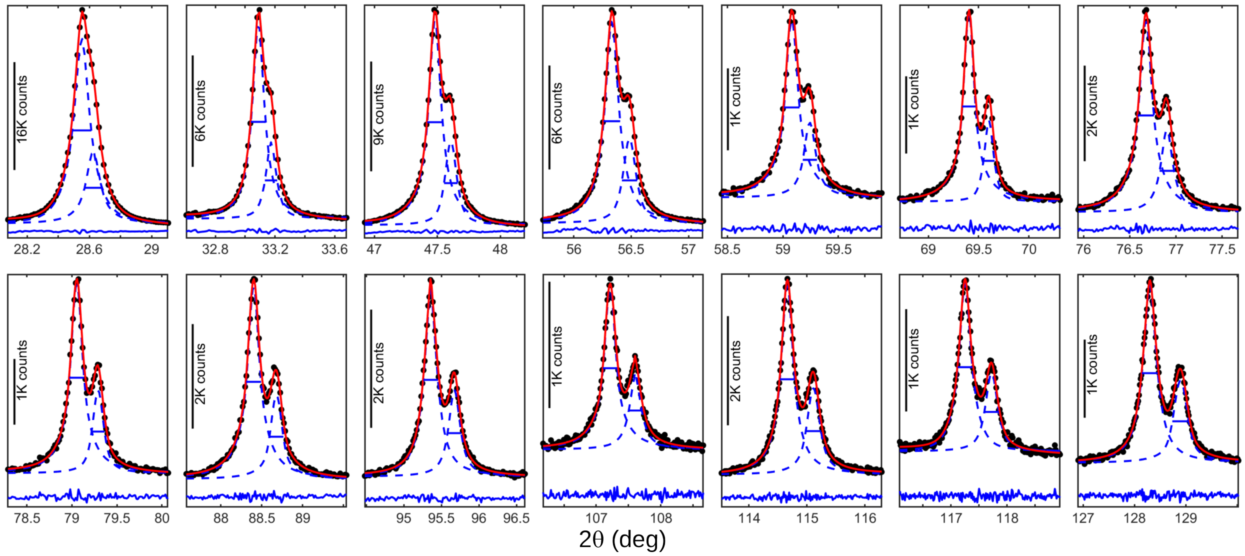

Figure S5: Sample B5. Line profile fitting (solid red lines) of $K_{\alpha 1}$ and $K_{\alpha 2}$ Bragg peaks (scatter dots) by using the pseudo-Voigt double function $V(2\theta)$, Eq. (S5). The FWHM of each $V_n(2\theta)$ function (dashed lines) are indicated by horizontal solid lines. The residuals of curve fitting are also shown (blue solid lines).

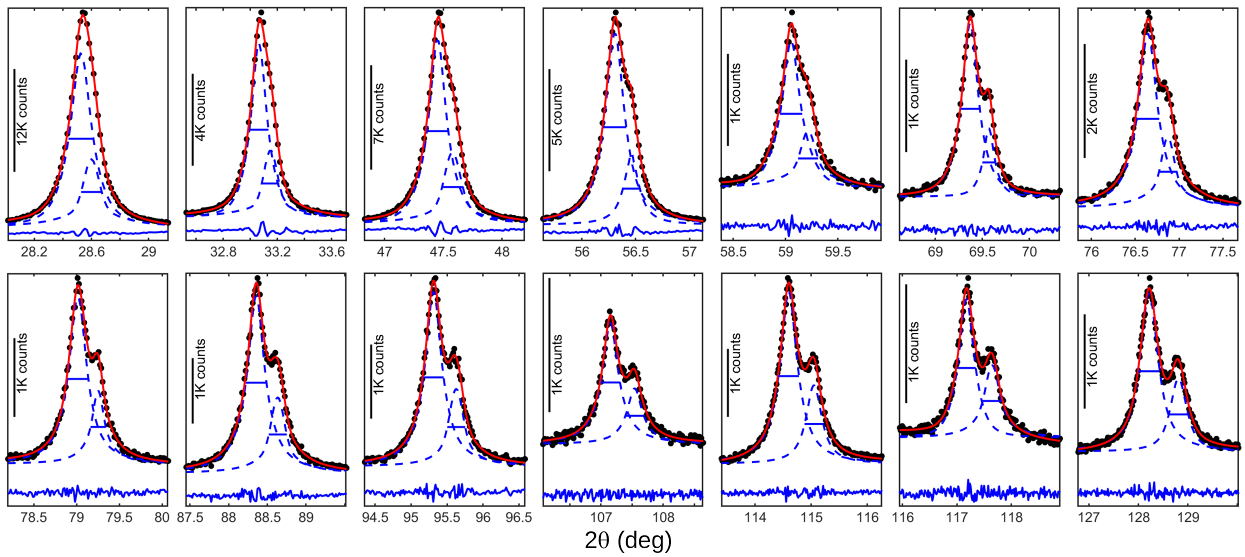

Figure S6: Sample B11. Line profile fitting (solid red lines) of $K_{\alpha 1}$ and $K_{\alpha 2}$ Bragg peaks (scatter dots) by using the pseudo-Voigt double function $V(2\theta)$, Eq. (S5). The FWHM of each $V_n(2\theta)$ function (dashed lines) are indicated by horizontal solid lines. The residuals of curve fitting are also shown (blue solid lines).

They were used to deconvolve instrumental broadening of the $\mathcal{W}_{\rm obs}$ values, leading to the instrumental free peak width $\mathcal{W}$ for the CeO_2 samples. For comparison purposes, Fig. S9(b) displays the corundum peak widths as obtained by whole pattern fitting.

Table S1: Crystallite sizes for CeO_2 samples as obtained by Rietveld refinement, Fig. S7(b-d). Cubic NPs, shape factor 0.86. Background: 8-coefficient Chebyschev polynomial. Fitting parameters: scale factor, detector zero, sample displacement, lattice parameter, size broadening (isotropic), Debye-Waller factors, and microstrain (isotropic). Instrumental parameters were fixed to the values obtained from the Al_2O_3 standard sample, Fig. S9(b).

sample	R_{wp}	reduced$-\chi^2$	microstrain (10^{-6})	crystallite size (nm)
C1	6.74%	3.75	487.2	102 ± 7
B5	7.07%	3.60	513.3	68 ± 5
B11	6.05%	2.66	999.7	54 ± 3

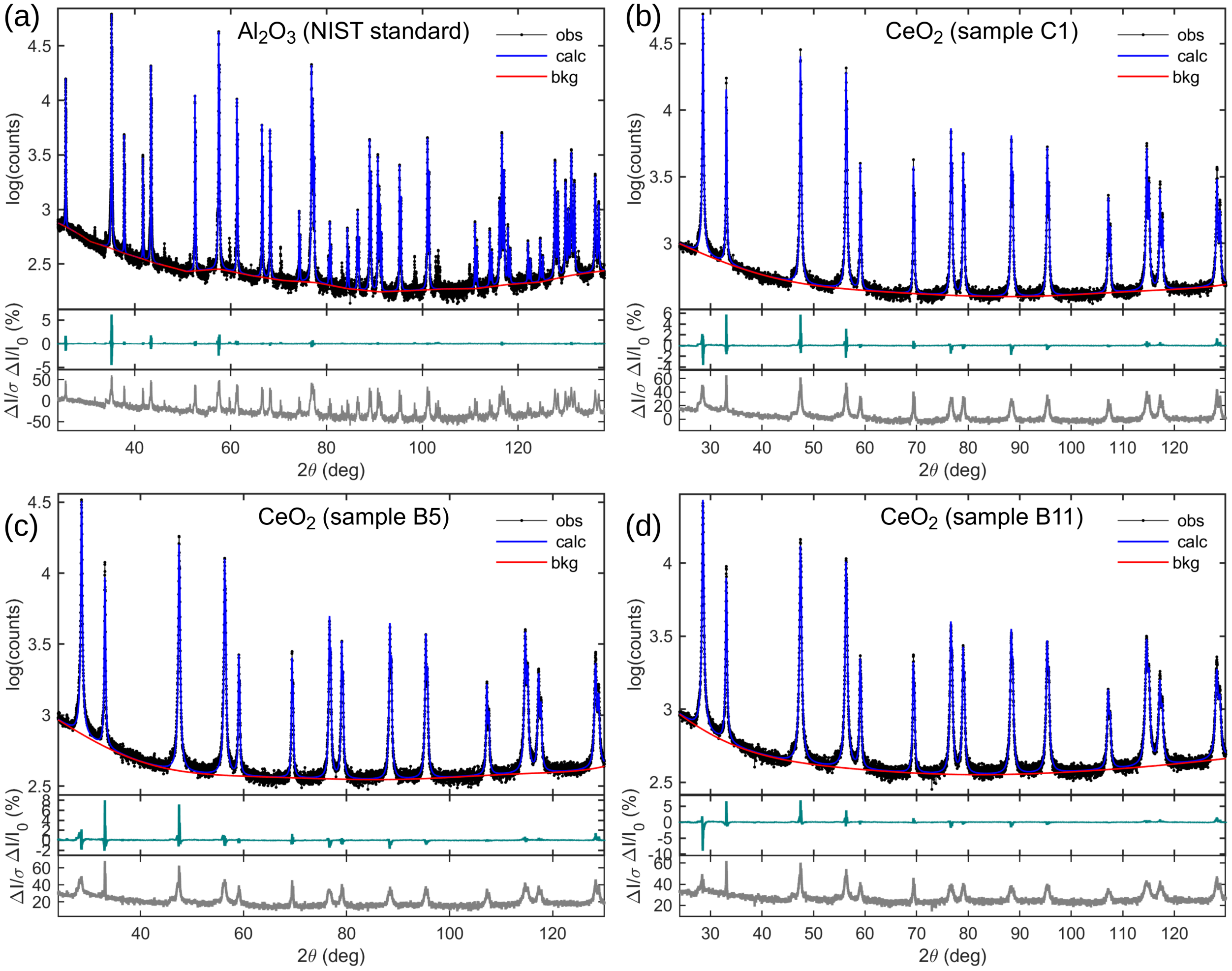

Figure S7: X-ray diffraction whole pattern fitting with GSAS-II. Experimental data (obs), calculated pattern (calc), and adjusted background (bkg). (a) NIST corundum standard 676a at room temperature.[8] (b-d) CeO_2 samples C1, B5, and B11, as indicated. Data collected in a D8 Discover Bruker diffractometer. CuK_α radiation (Ni filter). See Table S1 for adjustable parameters and fitting results.

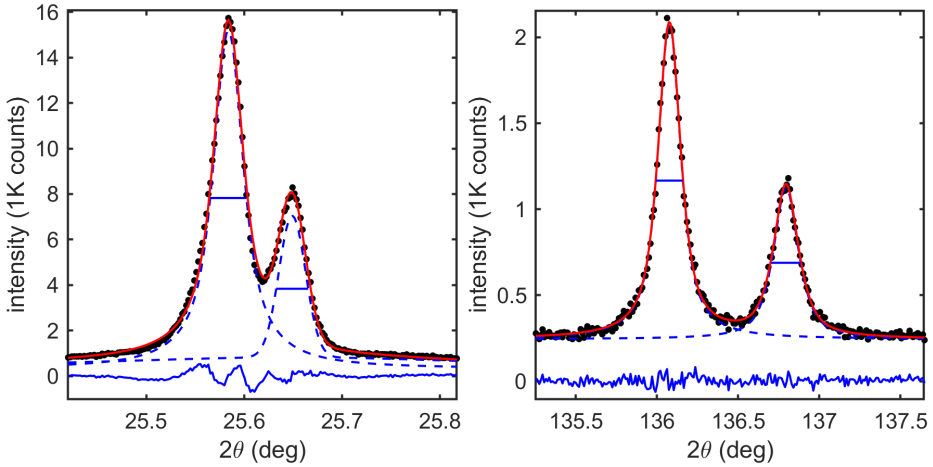

Figure S8: Examples of line profile fitting (solid red lines) of $K_{\alpha 1}$ and $K_{\alpha 2}$ peaks (scatter dots) by using the pseudo-Voigt double function $V(2\theta)$, Eq. (S5). The FWHM of each $V_n(2\theta)$ function (dashed lines) are indicated by horizontal solid lines. The residuals of curve fitting are also shown (blue solid lines). Both panels display Bragg reflections from the corundum pattern in Fig. S7(a).

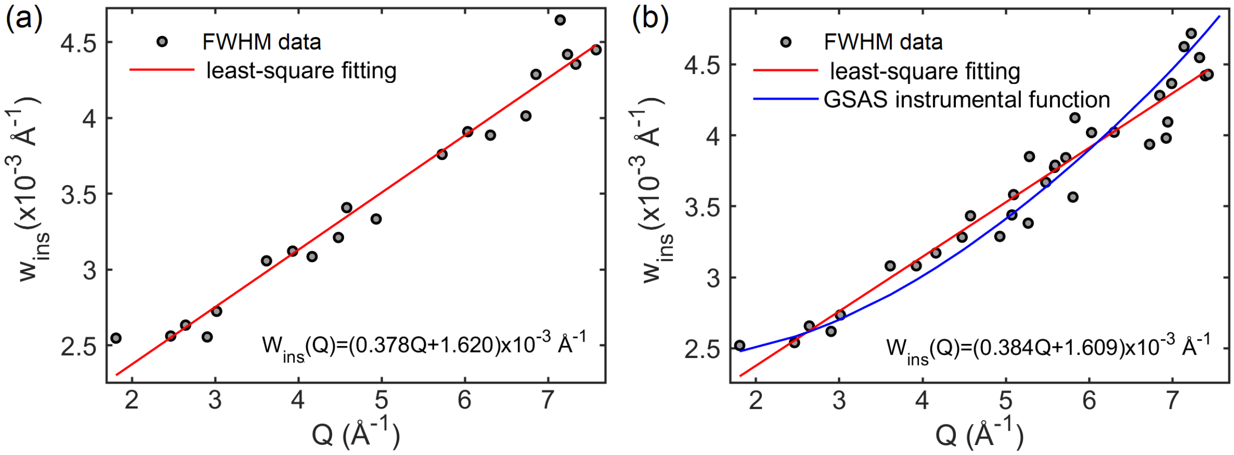

Figure S9: (a,b) Bragg peak width (scatter points) as a function of $Q = (4\pi/\lambda_1)\sin(\theta)$ of the standard sample in Fig. S7(a). Least-square fitting (solid red lines) of the data points leads to the instrumental width $\mathcal{W}_{\rm ins}(Q)$ as given in each plot. (a) From individual peak fitting with the pseudo-Voigt double function, Eq. (S5), and (b) with GSAS-II. Peak widths obtained by adjusting instrumental and size-broadening parameters available in the GSAS-II are also displayed (solid blue line).

3 WEIGHTED LOGNORMAL FUNCTION

In the particular case of a lognormal PSD function

$$n(k) = \frac{1}{k\sigma\sqrt{2\pi}} \exp\left[-\frac{\ln^2(k/\tilde{k})}{2\sigma^2}\right], \tag{S7}$$

the median value $\widetilde{K}_m$ of the k^m-weighted PSD is obtained as follow.

Integral solution by variable substitution is applicable for the integral

$$A_1 = \int_0^{\widetilde{K}_m} k^m n(k) dk = \frac{1}{\sigma\sqrt{2\pi}} \int_0^{\widetilde{K}_m} k^{(m-1)} \exp\left[-\frac{\ln^2(k/\tilde{k})}{2\sigma^2}\right] dk \tag{S8}$$

by changing $u = \sigma^{-1}\ln(k/\tilde{k})$, $du = (k\sigma)^{-1}dk$, and the upper and lower integral limits by $\tilde{u} = \sigma^{-1}\ln(\widetilde{K}_m/\tilde{k})$ and $-\infty$, respectively. Then,

$$A_1 = \frac{\tilde{k}^m}{\sqrt{2\pi}} \int_{-\infty}^{\tilde{u}} \mathrm{e}^{(mu\sigma - u^2/2)} du = \frac{\tilde{k}^m \mathrm{e}^{m^2\sigma^2}}{\sqrt{2\pi}} \int_{-\infty}^{\tilde{u}} \mathrm{e}^{-\frac{1}{2}(u-m\sigma)^2} du\,. \tag{S9}$$

Substitution of variable can be applied one more time, as $a = (u - m\sigma)/\sqrt{2}$ and $da = du/\sqrt{2}$, for which the upper and lower limits become $\tilde{a} = (\tilde{u} - m\sigma)/\sqrt{2}$ and $-\infty$. It results in

$$A_1 = \frac{\tilde{k}^m \mathrm{e}^{m^2\sigma^2}}{\sqrt{\pi}} \int_{-\infty}^{\tilde{a}} \mathrm{e}^{-a^2} da = \frac{1}{2}\tilde{k}^m \mathrm{e}^{m^2\sigma^2}\left[\mathrm{erf}(\tilde{a}) + 1\right] \tag{S10}$$

where erf is the the error function.[9,10] As $\widetilde{K}_m$ stands for the median value, the integral

$$A_2 = \int_{\widetilde{K}_m}^{\infty} k^m n(k) dk = \frac{1}{2}\tilde{k}^m \mathrm{e}^{m^2\sigma^2}\left[-\mathrm{erf}(\tilde{a}) + 1\right] \tag{S11}$$

can be solve by the same procedure of A_1, and both integrals must be equal. $A_1 = A_2$ in Eqs. (S10) and (S11) is possible as long as $\tilde{a} = 0$, or $\tilde{u} = \sigma^{-1}\ln(\widetilde{K}_m/\tilde{k}) = m\sigma$. It implies that

$$\widetilde{K}_m = \tilde{k}\exp(m\sigma^2) = k_0 \exp[(m+1)\sigma^2]\,, \tag{S12}$$

as used in this work for the k^4- and k^6-weighted PSD. $\tilde{k} = k_0 \exp(\sigma^2)$ is the unweighted PSD median value written in terms of the PSD mode k_0 and standard deviation in log scale σ.

References

(1) Debye, P. Zerstreuung von Röntgenstrahlen. *Annalen der Physik* **1915**, *351*, 809–823.

(2) Scardi, P.; Gelisio, L. Vibrational Properties of Nanocrystals from the Debye Scattering Equation. *Scientific Reports* **2016**, *6*, 22221.

(3) Morelhão, S. L. *Computer Simulation Tools for X-ray Analysis*; Graduate Texts in Physics; Springer, Cham, 2016.

(4) MatLabCodes, 2016; Appendix B, pp. 213 and 246 of Ref. 3. https://link.springer.com/content/pdf/bbm%3A978-3-319-19554-4%2F1.pdf.

(5) Cabral, A. J. F.; Valério, A.; Morelhão, S. L.; Checca, N. R.; Soares, M. M.; Remédios, C. M. R. Controlled Formation and Growth Kinetics of Phase-Pure, Crystalline BiFeO3 Nanoparticles. *Cryst. Growth Des.* **2019**,

(6) Wormington, M.; Panaccione, C.; Matney, K. M.; Bowen, D. K. Characterization of structures from X-ray scattering data using genetic algorithms. *Phil. Trans. R. Soc. Lond. A* **1999**, *357*, 2827–2848.

(7) Toby, B. H.; Von Dreele, R. B. *GSAS-II*: the genesis of a modern open-source all purpose crystallography software package. *J. Appl. Cryst.* **2013**, *46*, 544–549.

(8) Cline, J. P.; Von Dreele, R. B.; Winburn, R.; Stephens, P. W.; Filliben, J. J. Addressing the amorphous content issue in quantitative phase analysis: the certification of NIST standard reference material 676a. *Acta Cryst. A* **2011**, *67*, 357–367.

(9) Glaisher, J. W. L. LIV. On a class of definite integrals.—Part II. *The London, Edinburgh, and Dublin Philosophical Magazine and Journal of Science* **1871**, *42*, 421–436.

(10) Andrews, L. C. *Special Functions of Mathematics for Engineers*, 2nd ed.; Society of Photo-Optical Instrumentation Engineers - SPIE, 1997; p. 110.
